# High-resolution non-contact measurement of the electrical activity of plants *in situ* using optical recording

**DOI:** 10.1038/srep13425

**Published:** 2015-09-03

**Authors:** Dong-Jie Zhao, Yang Chen, Zi-Yang Wang, Lin Xue, Tong-Lin Mao, Yi-Min Liu, Zhong-Yi Wang, Lan Huang

**Affiliations:** 1 College of Information and Electrical Engineering, China Agricultural University, Beijing 100083, China; 2 State Key Laboratory of Plant Physiology and Biochemistry, Department of Plant Sciences, College of Biological Sciences, China Agricultural University, Beijing 100193, China; 3 Key Laboratory of Agricultural information acquisition technology (Beijing), Ministry of Agriculture, Beijing 100083, China

## Abstract

The limitations of conventional extracellular recording and intracellular recording make high-resolution multisite recording of plant bioelectrical activity *in situ* challenging. By combining a cooled charge-coupled device camera with a voltage-sensitive dye, we recorded the action potentials in the stem of *Helianthus annuus* and variation potentials at multiple sites simultaneously with high spatial resolution. The method of signal processing using coherence analysis was used to determine the synchronization of the selected signals. Our results provide direct visualization of the phloem, which is the distribution region of the electrical activities in the stem and leaf of *H. annuus*, and verify that the phloem is the main action potential transmission route in the stems of higher plants. Finally, the method of optical recording offers a unique opportunity to map the dynamic bioelectrical activity and provides an insight into the mechanisms of long-distance electrical signal transmission in higher plants.

Electrical signals in plants originate in response to environmental stimuli and play an important role in many physiological activities. Extracellular recording^1^,^2^,^3^,^4^,^5^,^6^, intracellular recording^7^ and patch-clamping^8^,^9^ have been widely used to measure these signals. Most of the current knowledge of the electrical signals in plants was obtained using these methods. The two main electrical signals found in plants are action potentials (APs) and variation potentials (VPs). The AP is used for long-distance transmission signaling. Many physiological effects such as gas exchange, gene expression and leaf movement can be induced or regulated by APs in plants^10^,^11^,^12^. VPs are slower signals that are induced by damaging stimuli^13^,^14^ (for example burning and cutting). Both types of signals carry information to other cells, tissues and organs, where they trigger appropriate responses.

The transmission of an AP in the stem has been studied in several higher plants. The phloem is thought to be the main transmission route for APs^15^. Researchers have attempted recording APs in the phloem using metal electrodes or microelectrodes to verify this. However, the methods listed above can only detect the extracellular potentials of the cells around the electrode (using metal electrodes), or measure the transmembrane potential change of single cells (using microelectrodes). There are limitations in both extracellular and intracellular recordings in terms of being able to distinguish changes in the electrical potential from multiple cells or different tissues in the stem of a plant. There have been no reports of high-resolution recordings from multiple sites that are capable of describing the AP distribution or transmission in the stems of plants.

Optical recording methods using voltage-sensitive dyes (VSDs) may overcome the limitations of extracellular and intracellular recording and allow monitoring of the bioelectrical activity at multiple sites with high resolution. Using a photon detector such as a photodiode array or a charge-coupled device (CCD) camera, it is possible to record the potential changes of multiple cells or regions in the microscopic field of view simultaneously^16^,^17^. Since the first recording of animal cell membrane potentials by noninvasive monitoring in the 1970s^18^, optical recording methods have been widely used in the study of APs from the heart, neuron and brain slices^19^,^20^,^21^,^22^. Recently, this method was used to perform measurements in plants^23^,^24^. Using a confocal microscope and the VSD RH-414, Furch and co-workers recorded single-site electrical signals induced by heat stimulation in *Vicia faba* L. and tomato leaf veins with a sampling interval of approximately 1 min^24^. The results of Furch *et al*. suggest the possibility of recording the electrical activity *in vivo* in plants using a VSD. However, the frame rate used was approximately 1 frame/min, which is not fast enough to record an AP. The VSD bis-(1,3-dibutylbarbituric acid)-trimethine oxonol (DiBAC_4_(3)) was found to be useful for probing the membrane potential change of guard cell protoplasts from *V. faba* L. induced by abscisic acid *in vitro*^25^. However, unlike in previous reports^23^,^24^, there are still challenges to imaging the electrical activity at multiple sites using VSDs *in situ* because of the cell walls of intact plants and problems with photobleaching.

To overcome these limitations, we developed a method to map the electrical activity induced by external stimulus in *Helianthus annuus in vivo in situ*. This method involved optical recording of electrical signals using DiBAC_4_(3), a VSD, to provide more direct evidence of whether the phloem is the main transmission route of the AP in the stems of higher plants. The optical recording method has a high spatial resolution that makes it possible to simultaneously measure the distribution of the electrical activity from different tissues *in vivo in situ* in a plant.

## Results

### Calibration of fluorescence intensity as a function of membrane potential *in situ*

To determine the reliability of this proposed method, we tested the baseline of optical recording *in situ* (Methods, Supplementary Figs. 1–2 and Supplementary Results) and compared the calibration results of protoplast *in vitro* (Methods, Supplementary Fig. 3 and Supplementary Results) to previous reports^25^,^26^. The calibration results of protoplast *in vitro* is similar to previous reports^25^,^26^. We next obtained stable baseline of optical recording for *H. annuus in situ* (Supplementary Figs. 1–2 and Supplementary Results).

**Figure 1:**
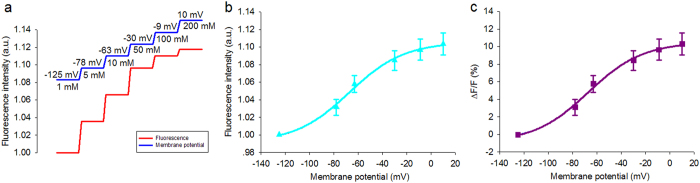
Dye calibration performed *in situ* in the *H. annuus* stem and leaf. (**a**) The dependence of the fluorescence intensity on the K^+^-induced membrane potential. The K^+^ concentrations used were 1 mM, 5 mM, 10 mM, 50 mM, 100 mM and 200 mM and the intracellular recordings by the microelectrode were approximately −125 ± 5 mV, −78 ± 4 mV, −63 ± 4 mV, −30 ± 4 mV, −9 ± 3 mV and 10 ± 1 mV (*n* = 25 recording repeats, 6 plant samples, mean ± S.E.M.) respectively. (**b**) The fluorescence ratio, Fc/F, versus the K^+^-induced membrane potential (*n* = 6 plant samples, mean ± S.E.M.). (**c**) The dependence of the normalized fluorescence, ΔF/F, and the membrane potential derived from (**b**).

For calibration of DiBAC_4_(3) *in situ*, the bath K^+^ concentration was increased from 1 mM to 5 mM, 10 mM, 50 mM,100 mM and 200 mM. The membrane potential was measured using a microelectrode without distinguishing the cell type. More than three sites in stem were selected (*n* = 25 recording repeats from six plant samples). The fluorescence images were acquired simultaneously from intact samples in a buffer solution with the different K^+^ concentrations at a pH of 6.5. The typical K^+^-induced raw fluorescence images recorded *in situ* are shown in Supplementary Figure 4. The measurement of the membrane potentials induced by extracellular K^+^ versus intracellular recording *in situ* using a microelectrode is shown in the upper trace of Fig. 1a. In Fig. 1a, the cell membrane potentials with different K^+^ concentrations were determined *in situ* (1, 5, 10, 50, 100 and 200 mM) were −125 ± 5 mV, −78 ± 4 mV, −63 ± 4 mV, −30 ± 4 mV, −9 ± 3 mV and 10 mV ± 1 mV respectively (*n* = 25 recording repeats, 6 plant samples, mean ± S.E.M.). After subtracting the background signal, the ratio of the fluorescence (Fc) at different K^+^ concentrations to the initial fluorescence intensity (F0) when the extracellular K^+^ concentration was 1 mM was measured. ΔF/F was calculated using (Fc−F0)/F0. The ratio Fc/F0 was dependent on the membrane potential (Fig. 1b). A plot of ΔF/F versus the membrane potential is shown in Fig. 1c. As shown in Fig. 1b,c, the fluorescence ratio Fc/F0 (or ΔF/F) changed in response to the membrane potential induced by extracellular K^+^. Between −125 mV and −100 mV there was low sensitivity of the signal. Between −100 mV and 0 mV, the ratio Fc/F0 (or ΔF/F) increased with increasing membrane potential. When the membrane potential was greater than 0 mV, the fluorescence did not change significantly with an increase in membrane potential. The calibration results show that ∆F/F was about 1% when the membrane was depolarized by 10 mV (Fig. 1c).

### Imaging the propagation of the electrical activity *in situ*

For *H. annuus*, electrical stimulus is considered to be an effective but non-damaging way to trigger an AP. AP characteristics recorded using the conventional extracellular method have been previously studied^11^. In this study, a 5 min recording using an Ag/AgCl electrode indicated that the stem potential was stable after *H. annuus* was rested for several hours (Supplementary Fig. 5a). The Ag/AgCl electrode did not record any APs before the electrical stimulus was applied (*n* = 20 samples). After electrical stimulation, the Ag/AgCl electrode recorded either a series of APs (Supplementary Fig. 5b,c) or a single AP (Supplementary Fig. 5d–f) in different samples. The amplitudes of the extracellular APs were about 30 mV because of the recording and reference electrode positions. The recording electrode was inserted into the stem and the reference electrode contacted the bath solution. The distance between the Ag/AgCl recoding electrode and reference electrode was about 0.5 cm.

**Figure 2:**
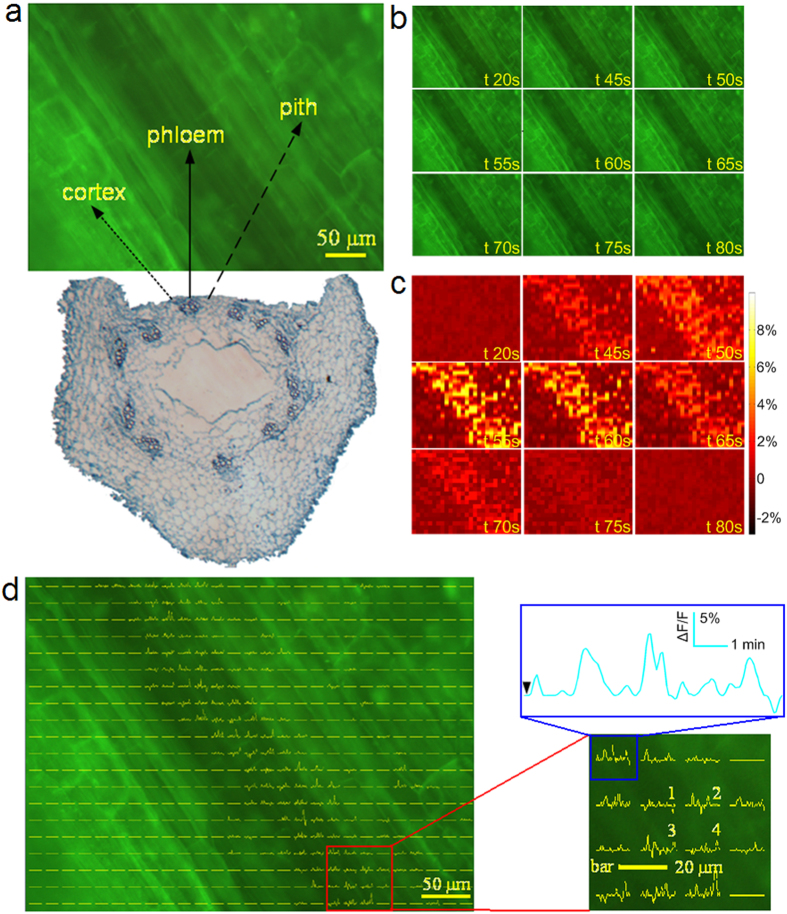
Typical optical recording of the distribution of the APs in the phloem region. (**a**) The different tissues such as the cortex, phloem and pith are shown in the radial face paraffin sections made at the end of the experiment. (**b**) A series of consecutive raw fluorescence images. (**c**) The time lapse ∆F/F images, where the variation of the fluorescence intensity can be clearly observed. (**d**) The results of the optical recording. The ΔF/F curves from the cortex, phloem and pith indicate that the ΔF/F change appears in the phloem region and that there was no obvious signal in the cortex and pith regions. The maximum amplitude of the ΔF/F signal is shown in the blue rectangle, which was magnified by approximately 8%. In the adjacent regions numbered 1, 2, 3, and 4 with center distances of about 20 μm, the maximum amplitude of the ΔF/F signal was about 5%, 7%, 10% and 4%, respectively.

**Figure 3:**
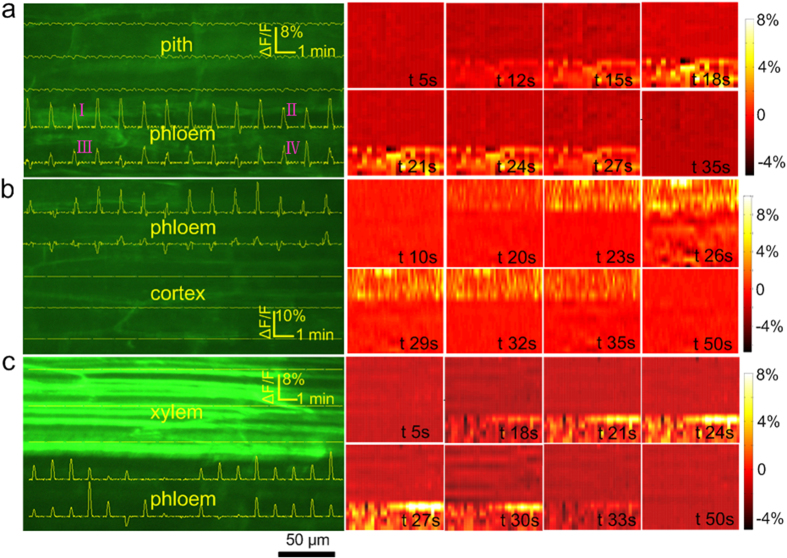
Typical optical recording of a single AP. The AP was distributed in the phloem region. (**a**) The ΔF/F curves are plotted on the left side. Each curve represents the change in the fluorescence intensity of the region around it with a spatial size of about 20 μm × 20 μm. From the signal curves numbered I, II, III and IV, similar ΔF/F waveform and amplitude can be observed, and in the whole region, the maximum amplitude of the ΔF/F signal is about 8%. On the right side, the time lapse ∆F/F images, are presented. The stimulus was applied at the fifth second. Following the stimulus, the ∆F/F change can be clearly observed in the phloem region. (**b**,**c**) The ∆F/F curves and the time lapse ∆F/F images of the other two samples. All of the images indicate that the AP is mainly distributed in the phloem region.

In our experiments, 132 *H. annuus* plants were measured. The Ag/AgCl electrode recorded the APs. The phloem region was observed in 20 samples. In these samples, the phloem was shown to be the main distribution region for the APs. However, there were no obvious signals in the cortex, pith or xylem regions. Figure 2 shows the results of the optical recording from one *H. annuus* sample in which the APs were induced using electrical stimulus applied at 9 V for 2 s (Supplementary Fig. 5b). By careful examination of radial face paraffin sections of the stems after recording, the cortex, the phloem and the pith could be identified as shown in Fig. 2a. Raw time lapse fluorescence images are shown in Fig. 2b, but it is hard to see the variation of the raw fluorescence intensity by the naked eye. This is because the amount of fluorescence change was small. However, in the time lapse ∆F/F pseudocolor images, the fluorescence variance (∆F/F) can be clearly observed and the electrical activity was distributed mainly in the phloem region (Fig. 2c). The ΔF/F curves are plotted in Fig. 2d. Each curve represents the fluorescence change of the region around it. The blue rectangle has a spatial size of 20 μm × 20 μm. The maximum of the ΔF/F curve in the blue region was 8% and the duration of the ΔF/F peaks is in tens of seconds. This clearly shows that the ΔF/F changes appear in the phloem region over the whole recording time period. The optical recording showed that the electrical activity induced by the stimulus could be represented using the ΔF/F changes when either a series of APs or a single AP was induced (Figs. 2, 3 and Supplementary Figs. 6,7).

**Figure 4:**
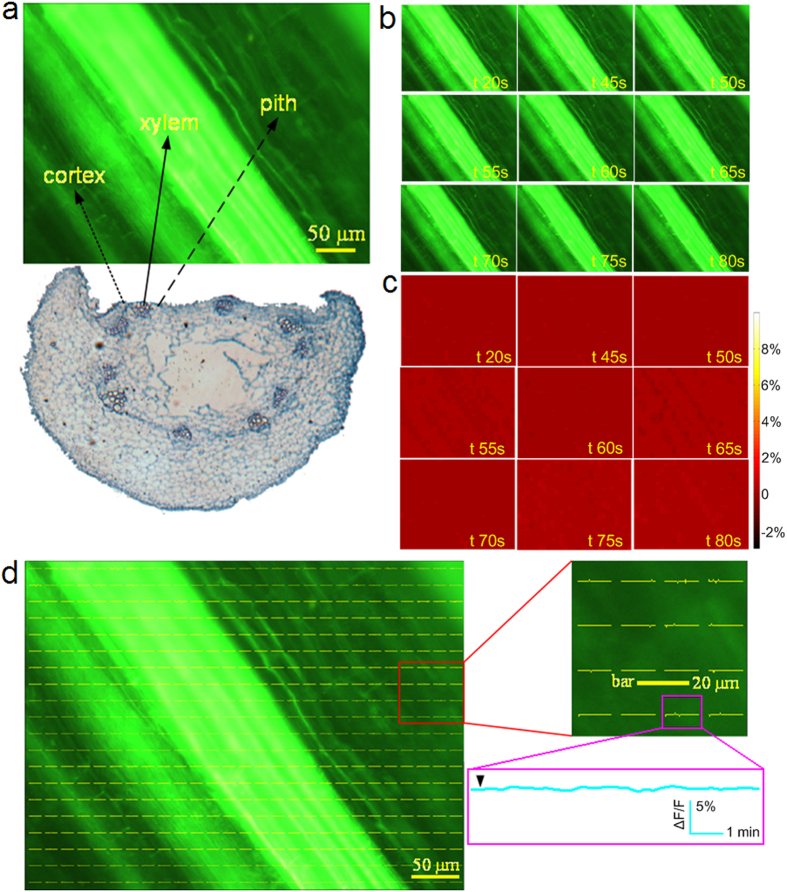
Optical recording of different tissues such as the cortex, xylem and pith. (**a**) An image of a radial face paraffin section that was made at the end of the experiment showing different tissues such as the cortex, xylem and pith. (**b**) A series of consecutive raw fluorescence images. (**c**) Time lapse ∆F/F images. No variation in the fluorescence intensity was observed. (**d**) The results of the optical recording. There was no ΔF/F change in the cortex, xylem or pith tissues, although the APs were recorded using the Ag/AgCl electrode.

Figure 3 shows the recordings of a single AP with the Ag/AgCl electrode (Supplementary Fig. 5d–f). From the ΔF/F curves on the left side and the time lapse ∆F/F images on the right side of Fig. 3a–c, the distribution of the APs can be clearly seen. Figure 4 shows the optical recordings of the cortex, xylem and pith tissues. The ΔF/F curves were plotted using the same method as that used in Figs. 2 and 3. Although the APs were recorded using the Ag/AgCl electrode across the stem (Supplementary Fig. 5c), no change in ΔF/F was found in the selected region where all three kinds of tissue (cortex, xylem and pith tissues) were present (Fig. 4c,d). The same results were found in the other samples (experimental data are not shown).

In higher plants, the phloem is considered to be the main transmission route for APs. Researchers have preliminarily verified this concept using microelectrode measurements combined with the injection of dye Lucifer Yellow solutions into cells to identify specific cell types^27^ and other conventional electrophysiological methods^24^,^28^. When compared with the conventional microelectrode method, our results produced a signal image with higher spatial resolution, in which the electrical characteristics from different tissues can be directly observed. The method provides direct visualization of APs in the phloem. Our results further verified that the phloem is the main AP transmission route in the stems of higher plants.

To test whether VPs could be detected *in situ*, simultaneous optical recording and conventional intracellular recordings (n = 4 plants) using microelectrode were performed, and then fluorescence was used to map the bioelectrical signals of the leaf or stem evoked by heat stimulation. Mapping of the heat-induced VPs in the petiole of the leaf or stem is also shown in Supplementary Fig. 8 and Supplementary Fig. 9. The fluorescence ratio ΔF/F (blue line) was scaled to overlay the intracellular recording (red line) (Supplementary Fig. 8b and Supplementary Fig. 9c).

### Coherence analysis of synchronized electrical signals in phloem

**Figure 5:**
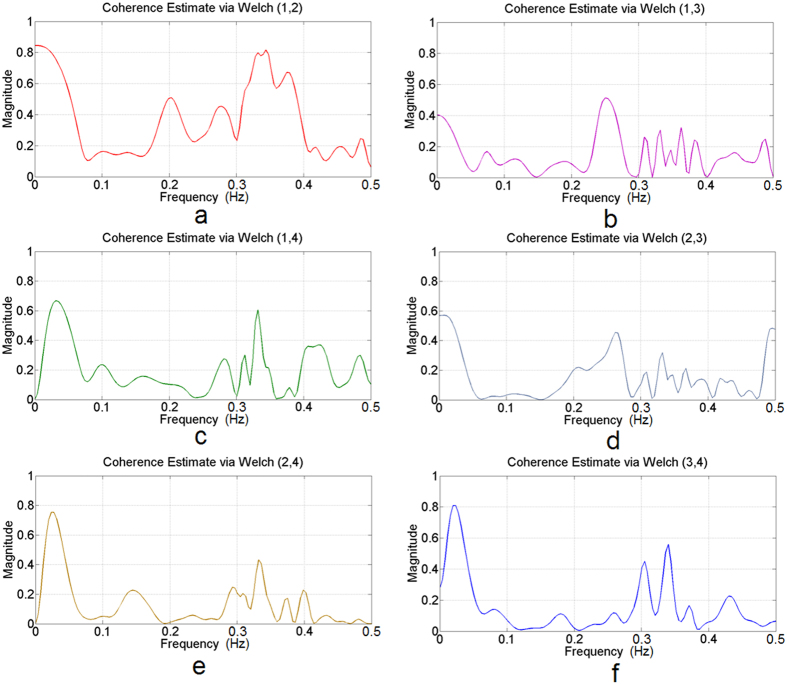
Results of coherence analysis for the different signal regions numbered on the right side of Fig. 2d. (**a**) Frequency of coherence between the numbered regions 1 and 2. (**b**) Frequency of coherence between the numbered regions 1 and 3. (**c**) Frequency of coherence between the numbered regions 1 and 4. (**d**) Frequency of coherence between the numbered regions 2 and 3. (**e**) Frequency of coherence between the numbered regions 2 and 4. (**f**) Frequency of coherence between the numbered regions 3 and 4. The maximum coherence magnitude (Coh_max_) of the signals is nearly 0.8 between signals 1 and 2 in (**a**). A signal of this magnitude appears in the low frequency range, which is lower than 0.1 Hz. (**b–f**) The Coh_max_ values between the different signals are nearly all higher than 0.5, and also appear in the frequency range below 0.1 Hz. Coherence analysis showed that there were high correlations (Coh_max_ > 0.5, *P* < 0.005, *t*-test) in the frequency domain between the different signal regions.

Although some features of the electrical signals in higher plants (for example maize) such as the power spectrum have been presented using a signal processing methodology^2^, the limitations of conventional extracellular recording and intracellular recording technologies mean that no studies have been conducted on the synchronization of AP transmissions in the phloem until this report. The phloem is a symplastic region connected by plasmodesmata. We simultaneously recorded potential changes at multisite from the phloem using the optical method. This provided data to support the synchronization analysis of the multiple site signals in the phloem. In our experiments, the maximum frequencies of the AP from Supplementary Fig. 5 that were recorded in the *H. annuus* stem by extracellular recording based on the fast Fourier transform were approximately 0.3 Hz and 0.25 Hz (Supplementary Fig. 10), respectively. In our study, the plant signals are generally low frequency signals of less than 1Hz. Therefore, coherence analysis can be used to collect sufficient information below 1 Hz. To reduce noise, the coherence analysis results of the ΔF/F curves were preprocessed with a smoothing method was described in the supplementary user guide for the software. Figure 5 compares the coherence analysis results of the ΔF/F curves numbered 1, 2, 3 and 4 that refer to the four different sites on the right side of Fig. 2d. Figure 5a shows that the maximum coherence (Coh_max_) values of signals 1 and 2 were nearly 0.8. A signal of this magnitude is in the low frequency range, which is lower than 0.1 Hz. In Fig. 5c–f, the maximum magnitudes are greater than 0.5 and also appear in the frequency range below 0.1 Hz. Only the coherence results for signals 1 and 3 shown in Fig. 5b had a small magnitude. The coherence analysis showed that there were high correlations (Coh_max_ > 0.5, *P* < 0.005, *t*-test) in the frequency domain between the different signal regions. In all other cases, similar results were observed (Supplementary Fig. 11). The analysis also suggested that the APs in the phloem of the *H. annuus* stem may be synchronized and that there may be a complex information transfer mode in higher plants. This result provides strong evidence that there are low-resistance bridges that allow AP transmission from cell to cell.

## Discussion

In this study, we used the VSD DiBAC_4_(3), which has a low toxicity^29^ and a slower response time (in hundreds of milliseconds) compared with fast response VSDs. Although this dye has limitations when recording fast electrical activity, our results show it can be used to record both APs and VPs in *H. annuus*.

The dye calibrations were performed both with protoplasts extracted from the *H. annuus* stem and *in situ* in *H. annuus*. For the protoplasts, the results agreed with those from the calibration of plant guard cell protoplasts^25^ and human embryonic kidney (HEK) 293 cells^26^. DiBAC_4_(3) is an anionic voltage-sensitive oxonol dye, which can enter depolarized cells and bind to intracellular proteins or membranes. It then exhibits enhanced fluorescence. The intracellular DiBAC_4_(3) has a partial negative charge and undergoes a Nernstian-like redistribution upon a membrane potential change^30^. The relationship between ΔF/F values and corresponding membrane potentials fit a sigmoidal function between −100 mV and 20 mV (determination coefficient *R*^2^ = 0.99) (see Supplementary Fig. 3d,e). The fluorescence change of the protoplasts was almost 1% per 1 mV and is consistent with previous reports^25^,^26^.

In the *in situ* calibration, the relation between ∆F/F values and the corresponding membrane potentials fit the sigmoidal function between −100 mV and 20 mV (determination coefficient *R*^2^ = 0.99) (Fig. 1b,c). Since the membrane potential is possibly associated with changes in pH, we examined the change in the dye properties with variation of the pH to exclude pH artifacts. When the pH was changed from 5.5 to 7.0 using the buffer solution, ∆F/F did not change significantly (Supplementary Fig. 12). The maximum deviation of ∆F/F of 0.68% ± 0.05% was similar to the noise level of the optical recording. In the *in situ* measurements, both electrical activities (APs and VPs) and changes in the pH were monitored simultaneously (Supplementary Fig. 13). The test results showed that pH changes in the buffer bath was smaller than 0.1 at 26 °C (n = 9 plant samples). Although the protoplasmic pH and cytoplasmic pH changes during an AP or VP in plants^31^,^32^, a small change in the pH had no significant influence on ∆F/F (Supplementary Figs. 12 and 13).

In the *in situ* calibration, the microelectrode recording was consistent with the change in membrane potential at each extracellular K^+^ concentration, which was calculated based on the Nernst equation. However, our findings indicated that the fluorescence change was almost 1% per 10 mV during *in situ* calibration. There were consistent findings from the calibration of the fluorescence intensity versus the membrane potential induced by extracellular K^+^ in the leaves (*n* = 3 plant samples) and stems (*n* = 6 plant samples) *in situ*. Although DiBAC_4_(3) was previously used to measure the plasma membrane potential of protoplasts, the loading of the dye for *in situ* recording presents unique challenges because the plant cell walls are intact and there is fluorescence scattering from multilayered cells. The *H. annuus* stem plasmolysis indicated that there was fluorescence in the membrane and even higher fluorescence in the cell wall region (Supplementary Fig. 14). Unlike the protoplast, plasmolyzation results suggested that the fluorescence signal may consist of two parts: emission from the cell wall and the cell membrane. The ratio of the emission from the cell wall and the cell membrane (n = 16 cells; Supplementary Figs. 14 and 15) was approximately 4:1; however, only the fluorescence emission from cell membrane was in response to a variation of the membrane potential. Together, these observations suggest that ∆F/F is smaller *in situ* than in protoplasts because the cell wall produces fluorescence that does not change when the membrane is depolarizing or repolarizing. Also, the fluorescence is scattered and reduced by the cell walls^33^,^34^. Therefore, the amplitude sensitivity was reduced from 1% per 1 mV to 1% per 10 mV *in situ*. Although *in vivo* observation of the membrane potential *in situ* is more complicated than in isolated protoplasts, the mapping propagation with high resolution using the VSD was nearly unaffected.

A major finding of this study was that extraction of data using the proposed method can be used to analyze the electrical signals from raw fluorescence images *in situ* effectively during photobleaching. This method used a common fluorescence microscope and continuous exposure to the excitation light. Photobleaching is an unwanted and inevitable dynamic process in which the fluorophore loses emission intensity from each excitation-emission process because of continuous exposure to the excitation light^35^. The original signals extracted from the raw fluorescence time lapse images indicated that the fluorescence intensity was a function of the rate of photobleaching (Supplementary Fig. 16). As shown in the images, when the fluorescence decreases the contrast is reduced, making it more difficult to distinguish genuine fluorescence changes by the naked eye^36^. To overcome the problem and recover a signal, a photobleaching correction algorithm was developed. The proposed method is similar to previous work on recordings from animal or human cells^37^ and modified. Considering its practical use in plants *in situ*, a single and double exponential curve fit was performed and statistical analysis of all curves was estimated. The fit was assessed by the evaluating the determination coefficient, *R*^2^, and the sum of squared errors, SSE.

It is worth noting that the fluorescence intensity decreased with time because of photobleaching. Using a photobleaching correction algorithm and pseudocolor images of ∆F/F, we could visualize the fluorescence change within the phloem tissue cannot be seen within the raw fluorescence images as shown in Fig. 2b. Based on the proposed method, real electrical activation changes that were superimposed on a decrease in fluorescence intensity were recovered. Thus, we can verify that the APs are transmitted through the stem and the range that the waves spread could be visualized for any selected site in the optical recording region, as shown in Fig. 2c,d and Supplementary Figs. 6 and 7.

The results shown in Figs. 2d, 3a–c and 4 indicate that APs only appear in the phloem and that there are no APs in the cortex or pith. In the xylem, the fluorescence was higher than in the other regions (Figs. 3c and 4). In vascular plants, the xylem participates in water transport and fills up with more dye, which was dissolved in the water-soluble dimethyl sulfoxide (DMSO) to stain the xylem cells. There are still many developing and immature vessel elements with membranes in the xylem loaded with DiBAC_4_(3). Also, a number of cells in the xylem are in the process of cell senescence, increasing the membrane potential. Evidence from cells in the process of extracellular adenosine triphosphate (ATP)-triggered programmed cell death indicated more pronounced membrane depolarization when using DiABC_4_(3)^38^. These factors may have resulted in the higher fluorescence intensity in the xylem when using DiBAC_4_(3). Although the fluorescence was higher, no AP-induced fluorescence intensity change was found in the xylem (Figs. 3c and 4).

We observed that there are differences between the traces shown in Figs. 2 and 3 and those in supplementary Fig. 5, which is likely because of the reasons in supplementary results. Thus, although there are differences between the ΔF/F profiles themselves, or between the ΔF/F profiles and the extracellular recordings of the APs, the optical recording can map these differences in a manner that traditional electrophysiological methods cannot. While the method presented here can map the distribution of the plant electrical activity with high spatial resolution, the analysis method used accurately links the spatial and temporal relationships between the membrane potential and the fluorescence from the overlapping layers of cells, and is still worthy of in-depth study in the future.

Our results showed that the optical method provides a more complete description of the dynamics of the global network of electrical activity in plants. Using optical recordings, we showed with high spatial resolution mapping that APs were generated by electrical stimuli and propagated in the stem of *H. annuus in vivo*. Here, we introduce a coherence analysis approach for characterizing synchronization at the low-frequency (<0.1 Hz) of electrical network activity. The maximum value in coherence analysis represents the highest synchronization level of the signals at a specific frequency. If the coherence coefficient is high, this suggests that a high level of synchronization exists in the signals, and indicates the possible transduction of signals. These findings help link the other interesting results of investigating synchronization using extracellular multielectrode array recording^39^. In general, the frequencies of plant electrical signals range from very low frequencies to several hundreds of Hz^4^,^40^,^41^,^42^,^43^, and they depend on the species of plant, the tissue measured, the growth stage of the plant, and the stimulation used (invasive or non-invasive stimulation). In our experiments, the maximum frequencies of the AP that were recorded in the *H. annuus* stem by extracellular recording were approximately 0.3 Hz (Supplementary Fig. 10). In future studies, acquisition of fast APs could be performed using a high speed and low noise CCD, such as the RedShirtImaging system.

For the transmission of APs in higher plants, the low electrical resistance of sieve tubes make them ideal pathways for long-distance electrical signaling, and the presence of plasmodesmata among the phloem cells provides routes for the passage of electric current between cells^15^,^44^,^45^. Although there are only a few plasmodesmata at the interface between companion cells and phloem cortex cells, resulting in weak electrical coupling, these lateral plasmodesmata make electrical signaling from neighboring cells to the sieve elements/companion cells possible^15^,^44^. In the phloem, the sieve tubes may also act as low-resistance pathways for electrical signals via plasmodesmata in the lateral direction and provide strong electrical coupling via the sieve pores in the longitudinal direction. Our findings provide strong new evidence that the phloem provides a network that offers routes for AP propagation in plants. This study, which was designed to enable mapping of the electrical activity in plants, could not be related to previously demonstrated results of the ion behavior in phloem from other reports^46^,^47^,^48^. Combining multisite optical recording method and other advanced techniques to clarify ion mechanisms involved in electrical activity remains an open area of interest for future research.

The need for plant cell observation on a small scale (tens of microns) and fluorescence collection means that a microscope is required during optical recording. The observed area is therefore limited to the focal plane of the objective. It is challenging to balance efficient fluorescence collection and achievement of a large field of view. Microscope objectives typically offer a tradeoff between magnification and light-gathering capacity^49^. Selecting an appropriate objective lens allows us to achieve a balance between the limitations and advantages of optical recording. The frame rate was set to one frame or five frames per second. Although this is a lower sampling rate than used in most conventional recording methods, 1 Hz can measure the signal without distortion when considering that depolarization and repolarization of APs occurs on a time scale from 10 s to 20 s (Fig. 2d). The 5 Hz sampling rate may allow us to investigate propagation of APs in phloem *in situ* (Fig. 3). Also, both sampling rates (1 Hz and 5 Hz) were applied to measure the VPs using DiBAC_4_(3) (Supplementary Figs. 8 and 9). In this study, we also calculated propagation velocity from both extracellular recording and optical recording (see Supplementary Figs. 17 and 18, Supplementary Table 2, Supplementary results). It was found that propagation of the electrical activity occurs at a mean velocity in the range of 0.70 ± 0.10 mm/s to 1.75 ± 0.14 mm/s, consistent with previous reports,^13^,^50^,^51^,^52^,^53^.

Even though a filter was used after excitation to reduce the exposure time to minimize photobleaching, this method was a special case using a modified fluorescence microscope. In practical applications, most common fluorescence microscopes can be used with a continuous illumination source as a universal method to measure electrical activities using VSD. Therefore, there is still a considerable effect of photobleaching on ∆F/F. For this reason, we recorded fluorescent images with a common fluorescence microscope at frame rates of 1 Hz and 5 Hz and used an exposure times of 1 s and 0.2 s, respectively. This effectively represents constant illumination. The challenge of dye photobleaching occurred in our experiments, and is widely reported in the literature^54^,^55^. Fortunately, there is a dynamics equation that can quantitatively address the photobleaching problem^35^,^56^, allowing development of methods to correct and extract signals from optical recordings, despite the presence of photobleaching^35^,^56^,^57^. On that basis, we proposed an algorithm to extract the required signal automatically. While the ΔF/F *vs*. membrane potential characteristic was a sigmoid function, the membrane potential can be determined quantitatively within a specific voltage range based on these functions^25^,^26^,^58^. Thus, we were able to determine the value of the membrane potential using these calibrated sigmoid functions. Both our results and previously published studies indicate that when a standard calibration is used, the function of ΔF/F versus membrane potential can determine the change of the membrane quantitatively, whether the function is linear or non-linear. Therefore, although the data were affected by photobleaching, the normalized fluorescence signal, ∆F/F, corresponding to the electrical activity, was extracted using our proposed algorithm.

In this study, the APs induced by electrical stimuli in the stem of *H. annuus* and VPs induced by heat stimulation were recorded simultaneously using optical recording and extracellular recording methods. It was found that the optical method using the voltage-sensitive dye DiBAC_4_(3) could effectively detect the potential changes from different tissues in the stem and leaf of *H. annuus*. The analysis results also showed that the APs were distributed in the phloem of the *H. annuus* stem. The optical recording method with high spatial resolution introduced here can provide data for analysis of the relationships between different signal regions. With increasing instrument performance levels, it will be possible to apply the optical method using voltage-sensitive dyes much more widely in the study of electrical signals in plants.

## Online Methods

### Plant material and growth conditions

*H. annuus* plants were grown in a greenhouse under a 16 h light/8 h dark photoperiod regime with 30 °C day and 25 °C night temperatures. The growth medium comprised 70% vermiculite and 30% humus and contained all essential nutrients. The plants that were used for the experiments were 2–3 weeks old, with stem diameters of 2 mm to 2.5 mm.

### Voltage-sensitive dye solution and sample preparation

In this study, a potential sensitive probe DiBAC_4_(3) (Sigma-Aldrich) was used. The peak excitation and emission wavelengths of the dye are 485 nm and 515 nm, respectively. Increased depolarization results in an additional influx of the anionic dye and an increase in fluorescence. Conversely, hyperpolarization results in a decrease in fluorescence. The detailed characteristics of DiBAC_4_(3) have been described in the literature^25^,^26^. In this study, 25 mg DiBAC_4_(3) was dissolved in 1.212 mL DMSO and stored at 4 °C. Before preparation of the *H. annuus* sample, 30 μL dye was added to 120 mL base solution, which contained 0.5 mM CaCl_2_, 2.5 mM HEPES-NaOH (pH 6.5), 10 mM sucrose and 1.5 mM KCl (Sigma-Aldrich). The final concentration of DiBAC_4_(3) was 10 μM with approximately 0.025% DMSO.

To effectively load DiBAC_4_(3) to stem of *H. annuus in situ*, , two small parallel longitudinal incisions and a connecting horizontal incision with depths of less than 1 mm were made in the stem surface to prepare the samples. A strip was torn along the cambial interface using sharp tweezers and was cut off at approximately 5 mm long. The phloem or xylem was exposed on the tangential face with a suitably deep horizontal cut. To avoid a blurred image imaging from deep within thick tissues (see supplementary methods, supplementary results and supplementary Fig. 19), this was performed using a horizontal incision of approximately 0.44 mm when the stem diameter was about 2.5 mm. After the removal of this strip, the *H. annuus* stem was immediately immersed in the dye solution. The Ag/AgCl recording electrode and a pair of Pt stimulation electrodes (0.1 mm) spaced approximately 1.5 cm apart were inserted into the stem. The *H. annuus* plant was cultured for 2–5 h at room temperature (26–28 °C) in the dark. At the end of each experiment, the radial face of the stem at the cut location was made into a paraffin section and the different cells were identified and marked in the tangential face from observation of the paraffin section under a microscope (XDS-1B, ChongQing Optical & Electrical Instrument Co., Ltd., Chongqing, China).

### Preparation of protoplasts

Protoplasts were isolated from young *H. annuus* stems using a two-step procedure^25^,^59^. A 2 cm *H. annuus* stem segment was cut into 0.1–0.2 mm pieces in an enzyme solution composed of 1% Cellulase RS (w/v), 0.5% Macerozym R-10 (w/V), 1% Pectolyase (w/v), and 600 mM Mannitol (pH 5.7). Then, the 10 mL enzyme solution containing the stem debris was transferred to a shaker and shaken for 10 h at 50 rpm at 25 °C. Following the enzyme treatment, the protoplasts were washed though a 250 μm mesh. The filtered protoplasts were subjected to their first centrifugation at 100 g for 5 min. The supernatant was decanted and 5 mL of culture solution composed of 0.1% glucose, 0.08% KCl, 0.9% NaCl, 1.84% CaCl_2_ and 2 mM MES-KOH (pH 5.7) was added to the substrate. After a second centrifugation of 100 g for 5 min, the supernatant was decanted, and 5 mL of fresh culture solution containing 10 μM of DiBAC_4_(3) was added to the protoplasts. The protoplasts were stored at 4 °C for 4 h. Calibration was then performed at room temperature (26 °C).

### Dye calibration with protoplasts depolarized by changes in the K^+^ concentration

After isolation, the protoplasts were incubated in a buffer including 0.1% glucose, 0.08% KCl, 0.9% NaCl, 1.84% CaCl and 2 mM MES-KOH in the dark at 4 °C. Prior to calibration, 5 mL of fresh culture solution containing DiBAC_4_(3) was added to the protoplasts. The final concentration of DiBAC_4_(3) was 10 μM. According to methods in the literature^25^, different extracellular K^**+**^ concentrations can change the membrane potential of tissues or cells. In this experiment, K^**+**^ concentration in buffered solutions was increased stepwise from 1 mM to 5 mM, 10 mM, 50 mM, 100 mM and 200 mM by perfusion system (VC^3^ 4C, ALA Scientific Instruments, Inc., New York, USA) with 0.1% glucose, 0.9% NaCl, 1.84% CaCl, and 2 mM MES-KOH and 10 μM DiBAC_4_(3), pH = 5.7. When the solutions reached a steady desired final K^+^ concentration, the steady level was maintained, and then electrophysiological and optical recording was performed simultaneously at ∼3 min. The membrane potential of the protoplasts was measured using a microelectrode. After focusing on an appropriate region that contained many protoplasts in the bright field using a 20 × objective lens, the dye was excited and the fluorescence image was captured immediately. At each K^**+**^ concentration, the total exposure time was less than 2 s to minimize photobleaching. To reduce photobleaching, the excitation light was covered with a filter after image capture.

The measurements were then corrected for the background fluorescence recorded from reference regions close to the protoplasts under analysis. The fluorescence images of protoplasts with different K^**+**^ concentrations were recorded and ∆F/F was calculated.

### Dye calibration *in situ* in the *H. annuus* stem and leaf

A tiny incision in the stem or the leaf petiole of approximately 1 mm^2^ was exposed for staining with DiBAC_4_(3) in the buffer solution. After sample preparation, the *H. annuus* stem or leaf petiole was immediately immersed in a 10 mL buffer solution with the dye for 4 h. Here, we used different extracellular K^**+**^ concentrations to change the membrane potential of the tissues in the stem and leaf of *H. annuus*. K^**+**^ concentrations was increased stepwise from 1 mM to 5 mM, 10 mM, 50 mM, 100 mM and 200 mM by perfusion system (VC^3^-4C, ALA Scientific Instruments, Inc., New York, USA). The composition of the rest of the buffer solution remained the same and consisted of 0.5 mM CaCl_2_, 2.5 mM HEPES-NaOH (pH 6.5), 10 mM sucrose and 10 μM DiBAC_4(_3). When the solutions reached a steady desired final K^+^ concentration, the steady level was maintained, electrophysiological and optical recording was performed simultaneously. The intracellular recordings were measured with a microelectrode. For each K^**+**^ concentration, the total exposure time was less than 2 s to slow down the photobleaching. Then, the fluorescence images *in situ* with different K^**+**^ concentrations were recorded and ∆F/F was calculated from regions of interest (ROIs).

### Optical recording

**Figure 6:**
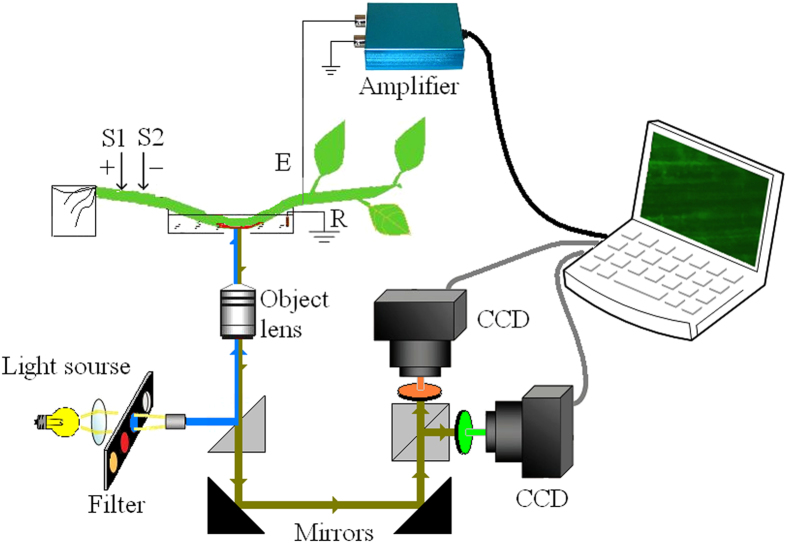
Optical recording system. Excitation light from a high pressure mercury lamp passed through a band pass excitation filter to irradiate the *H. annuus* stem immersed in the dye solution. The epifluorescence was passed through the green channel filter, collected by a 20 × NA = 0.40 objective, and then detected using a cooled CCD camera. The fluorescence image was displayed on the computer screen by the control software. The symmetrical design of the CCD camera layout satisfies the requirements of different dyes, but only the green channel CCD was used for DiBAC_4_(3). S1 and S2 are the stimulating electrodes using 0.1 mm Pt wires. S1 is the anode, E is the Ag/AgCl electrode and R is the reference electrode connected to the ground. This figure was drawn by Zhong-Yi Wang, and the fluorescence image shown in the computer screen was provided by Dong-Jie Zhao.

In this study, the optical recording system setup, as shown in Fig. 6, was built around an inverted fluorescence microscope (XSP-63XD, Beijing Sunguang Optical Instrument Co., Ltd., Beijing, China). The excitation light produced by a high pressure 100 W mercury lamp was collimated using an excitation filter that was centered at 488 nm and had pass bands of 20 nm (50% of the maximum transmission). Epifluorescence from the dye that passed through a 515 nm long-pass filter was detected using a cooled CCD camera (TCH-1.4CICE, Tucsen, Fuzhou, Fujian, China). The camera had a 1360 × 1024 pixel CCD, and the microscope used a 20 × NA = 0.40 objective lens. Thus, each pixel is corresponded to received light from a 0.32 × 0.32 μm^2^ area of the *H. annuus* stem. The total measurement area under the microscope was 0.435 × 0.328 mm^2^ using a 20 × NA = 0.40 objective lens. The CCD camera frame rate was set at one frame or five frames per second. The exposure time (1.0 s or 0.2 s) and the gain (400) were set using the camera’s control software (Tsview7, Tucsen). The mapped fluorescence images were also saved on the hard disk of a computer by the software. All experiments were conducted at room temperature (26 °C).

This figure was drawn by Zhong-Yi Wang, and the fluorescence images shown in the computer screen and the computer (K500A-B95 D1, Hasee Computer Co., Ltd., Shenzhen, Guangdong, China) were provided by Dong-Jie Zhao.

### Electrophysiology experiments

For the intracellular recording, a glass microelectrode with 500 mM KCl and Ag/AgCl was inserted into a cell in the stem, which was immersed in the bath solution using a micromanipulator (STW-3C, Chengdu Instrument Factory, Chengdu, China) beneath an inverted microscope (XSP-63XD, Beijing Sunguang Optical Instrument Co., Ltd., Beijing, China). The bath solution contained 0.5 mM CaCl_2_, 2.5 mM HEPES-NaOH (pH 6.5), 10 mM sucrose, 1.5 mM KCl and 10 μM DiBAC_4_(3). The reference electrode was located in the solution.

The extracellular recording of the APs used the Ag/AgCl electrode and the electrical stimulus (9 ± 2V, 3 ± 1 s) was applied to the stem through the Pt electrodes by a voltage stimulator (Chengdu Instrument Factory, Chengdu, China). The positions of the electrodes are shown in Fig. 6. The distances between the stimulating electrode S2 and the objective lens, and between S2 and the Ag/AgCl recording electrode are 10 mm and 20 mm, respectively.

The output voltage of intracellular recording or extracellular recording was passed through a high impedance (2 × 10^13^ Ω) operational amplifier (SWF-1B, Chengdu Instrument Factory, China) that was used as a voltage follower. The signal was then digitized stored in a signal acquisition system using custom-made software (RM6240, Chengdu Instrument Factory, China) and a computer hard drive. The plant was situated on an anti-vibration table in a Faraday cage (Inbio Life Science Instrument Co., China) in the dark.

### Signal extraction from the fluorescence images

Raw fluorescence traces were corrected for background fluorescence and photobleaching. The average gray value of selecting ROIs was extracted in turn from the fluorescence images, and then the original optical signal F(t) was obtained. When considering a universal method, consecutive exposure and recording using a general fluorescence microscope results in inevitable photobleaching during the course of recording the response to stimulation. In real experiments, because of continuous exposure to the excitation light, fluorescence changes in response to the electrical activity are superimposed on the decay of the fluorescence intensity. The fluorescence intensity decays exponentially with photobleaching^35^. There are different types of photobleaching curves, which are generally modeled using a mono-exponential or double exponential curve^35^,^37^. For practical purposes, the photobleaching curve at a stable membrane voltage of F0(t) was fitted to a single exponential function and a double exponential function before stimulation. The two fitting equations are given as Equation (1) and Equation (2), which represent a single exponential function and a double exponential function, respectively. In Equation (1), F0 is the initial intensity, *a* is the time constant (the rate of photobleaching) and *t* is time. In Equation (2), F01 and F02 correspond to initial intensity at the start of the fit, and *b* and *c* are two different rates of photobleaching.


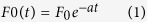



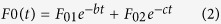


Statistical analysis allows the evaluation of the fit using the determination coefficient, *R*^2^, and the sum of squared errors, SSE. Based on the value of *R*^2^, a single exponential function or a double exponential function was determined and applied. Photobleaching was corrected for with the division of the exponential fit of the fluorescence trace, F0(t). Thus, ΔF(t) = F(t)−F0(t), where ΔF/F0(t) is the normalized change in the fluorescence intensities. In this study, the potential change of the cell membrane was determined by calculating ΔF/F (ΔF/F0(t)) for the series of fluorescence images. This is a general method used for quantification of the change in potential when using VSDs^60^,^61^.

Therefore, we proposed an algorithm to identify the changes of the signal induced by stimulation effectively despite the photobleaching. The main signal extraction steps were as follows. First, certain pixels were selected in a single frame. The average values of these pixels from the same region of every frame were selected as the time course data, F(t). To obtain the time dependence of the photobleaching, the first 100 sampling points before stimulation from F(t) and final 20 sampling points from F(t) were used to fit the trace using the exponent function form F0(t). Then, the ΔF/F curves of all the regions in the frame could be loop-computed and plotted in the frame. The detailed signal extraction flow can be seen in our previous work^23^. All calculations were performed using the custom-written software (Supplementary Software) based on Matlab (Matlab R2009a).

### Coherence analysis

Electrophysiological signals such as APs in plants can be regarded as the overlap of many signal components with different frequencies according to Fourier transformation theory. Coherence analysis is a statistical approach that can be used to reveal the degree of relationship or association between two signals at a particular frequency^62^. It is computed by normalizing the magnitude of the summed cross-spectral density between two signals by their respective power in the frequency domain. Its value is a number between 0 and 1 for each frequency. In this study, we used the magnitude squared coherence estimate, which indicates how well signal *x* corresponds to signal *y* at each frequency. The definition of the estimate is:


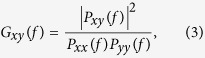


where *P*_*xy*_(*f*) is the cross power spectral density of signals *x* and *y*, and *P*_*xx*_(*f*) and *P*_*yy*_(*f*) are the power spectral densities for *x* and *y*, respectively. Computation of the coherence between the signals forming in regions of interest (ROIs) was performed using the Matlab coherence function based on Welch’s method^63^.

### Data analysis

Data are presented as the mean ± S.E.M. A Student’s t-test was used to analyze the high correlations in different signal regions, and p < 0.05 was considered significant.

## Additional Information

**How to cite this article**: Zhao, D.-J. *et al*. High-resolution non-contact measurement of the electrical activity of plants *in situ* using optical recording. *Sci. Rep*. **5**, 13425; doi: 10.1038/srep13425 (2015).

## Supplementary Material

Supplementary Information
