# Supplementary material for: High-resolution non-contact measurement of the electrical activity of plants in situ using optical recording
Source: Sci Rep. 2015 Sep 3;5:13425. doi: 10.1038/srep13425 (PMC4558603; doi:10.1038/srep13425)
Supplement: Supplementary Information [file srep13425-s1.pdf]

High-resolution non-contact measurement of the electrical activity of plants *in situ* using optical recording

Dong-Jie Zhao[1], Yang Chen[1], Zi-Yang Wang[1], Lin Xue[1], Tong-Lin Mao[2], Yi-Min Liu[2], Zhong-Yi Wang[1,3]* and Lan Huang[1,3]*

[1]College of Information and Electrical Engineering, China Agricultural University, Beijing 100083, China

[2]State Key Laboratory of Plant Physiology and Biochemistry, Department of Plant Sciences, College of Biological Sciences, China Agricultural University, Beijing 100193, China

[3]Key Laboratory of Agricultural information acquisition technology (Beijing), Ministry of Agriculture, Beijing 100083, China

*Corresponding authors. Z.Y.W. (wzyhl@cau.edu.cn) and L.H. (hlan@cau.edu.cn)

Inventory of Supplementary Information

**Supplementary Methods**
Supplemental Method 1 Fluorescence image analysis after plasmolysis
Supplemental Method 2 Heat stimulation and recording
Supplemental Method 3 Measurement of pH using ion selective microelectrode
Supplemental Method 4 Extracellular APs recording using a gold electrode
**Supplementary Results**
Stable baseline of optical recording for *H. annuus in situ*
Calibration of the fluorescence intensity versus the membrane potential *in vitro*
Spatial and temporal relation between membrane potential and fluorescence
Measurement of propagation velocity using both extracellular recording and optical recording
**Supplementary Note**
Supplementary Note 1
Supplementary Note 2

Supplementary Note 3   Compressed File instruction to readers
**Figure S1**
**Figure S2**
**Figure S3**
**Figure S4**
**Figure S5**
**Figure S6**
**Figure S7**
**Figure S8**
**Figure S9**
**Figure S10**
**Figure S11**
**Figure S12**
**Figure S13**
**Figure S14**
**Figure S15**
**Figure S16**
**Figure S17**
**Figure S18**
**Figure S19**
**Figure S20**
**Supplementary Table 1**
**Supplementary Table 2**
**Supplementary Table 3**
**Supplementary references**

Supplementary Methods

Fluorescence image analysis after plasmolysis

Plasmolysis was used to investigate the effect of loading the dye into the cell membrane with an

intact wall. The stained stem and epidermal strips were soaked in buffer solution including 0.5 mM $CaCl_2$, 2.5 mM HEPES-NaOH (pH 6.5), 10 mM sucrose, and 1.5 mM KCl, 10 μM $DiBAC_4(3)$ and 30% sucrose for 10 min at room temperature (26 ℃). The fluorescence images were recorded with a fluorescence microscope (XSP-63XD, Beijing Sunguang Optical Instrument Co., Ltd., Beijing, China) and a laser scanning confocal microscope (Confocal Leica SP5, Germany), respectively. When confocal microscope was used, the observations were performed without a coverslip using a 40× oil objective. $DiBAC_4(3)$ was excited at 488 nm and a band pass emission filter was used (515–590 nm) for observation.

The bright field and background autofluorescence images before staining with $DiBAC_4(3)$ were recorded using an excitation wavelength of 488 nm. After plasmolysis, we obtained fluorescence images of the stained stem and epidermal strips excited at 488 nm, respectively. A rectangular box of approximately 900 pixels in size was used to select an area of the cell wall and cell membrane from the same cell in the fluorescence image. The averaged raw fluorescence of the box was calculated by dividing the total fluorescence of the box after background correction by the total number of pixels in the box. In the image, three positions were numbered 1, 2 and 3 where the raw fluorescence F_{raw1}, F_{raw2} and F_{raw3} were obtained, respectively. The normalized fluorescence F1, F2 and F3 was then calculated using $F1 = F_{raw1}/F_{raw1}$, $F2 = F_{raw2}/F_{raw1}$, $F3 = F_{raw3}/F_{raw1}$. The fluorescence intensity of the cell membrane was calculated using $Fm = F1-F2$, where F1 is the fluorescence from both the cell wall and cell membrane, F2 is the fluorescence from the area at the top side of the wall and F3 is the fluorescence intensity from edge of the cell wall. F4 is the sum of F2 and F3 and does not contribute to the signal. When $\frac{\Delta Fm}{Fm+F4}$ is calculated, however, F4 does contribute to the resting fluorescence, which decreases the ΔF/F.

Heat stimulation and recording

In this experiment, a tiny incision of approximately 1 mm^2 in the petiole or stem was exposed for $DiBAC_4(3)$ staining in the buffered bath solution consisting of 0.5 mM $CaCl_2$, 2.5 mM HEPES-NaOH (pH 6.5), 10 mM sucrose, and 1.5 mM KCl, and 10 μM $DiBAC_4(3)$ for at least 1 h. Thermal stimulation was applied to a nearby leaf, which was separate from the recording leaf. Stimulation was performed by passing a flaming match under the leaf tip for 3 s in an area of approximately 3 mm^2. The distance between the stimulation point and the recording region (petiole) was 8–10 cm. Fluorescence

images were recorded using a common fluorescence microscope with a 10× NA = 0.25 objective lens at a frame rate of 5 Hz and an exposure time of 0.2 s. The fluorescence images and electrical signals in the adjacent leaf or stem were recorded simultaneously (Methods in manuscript). The fluorescence ΔF/F *in situ* was calculated from regions of interest (ROIs) after correcting the signal for photobleaching.

Measurement of pH using ion selective microelectrode

The preparation of ion selective microelectrodes and measurement of pH have been described in many previous reports[1-3]. Briefly, micropipettes were pulled from borosilicate glass capillaries without a filling fiber (TW150-3, World Precision Instruments, Inc., USA) by using a puller (P97, Sutter Instrument Company, USA). After pulling, the micropipettes with tip diameters of 3-4 µm were dried at 150℃ for 2h. Next, micropipettes was silanized using 5% dimethyldichlorosilane (Fluka Chemicals,UK) at 150℃ for 30 min. The silanized pipettes tips were filled with a 30 µm column of H^+-selective ionophore cocktails (95293, Fluka Chemical,UK) and backfilled with solution containing 500 mM KCl and Mes/Tris adjusted to pH 6.5. The pH-sensitive microelectrode and reference electrode were connected to the two-channel high-impedance ($2\times10^{13}\Omega$) amplifier (SWF-1B, Chengdu Instrument Factory, China) through Ag/AgCl wires. The Nernstian properties of each electrode were determined by three-point calibrations using a series of standard solutions (see Supplementary Table 1). The microelectrode tip was placed at approximately 4±1 µm from the tissue surface of a peeled region of the stem using a micromanipulator and then extracellular pH was measured.

Extracellular APs recording using a gold electrode

The stem of *H. annuus* was immersed in the dye solution after a 5–10 mm stripe was removed with sharp tweezers. The stain protocol has been described in the Methods. A pair of 0.1 mm Pt stimulation electrodes, spaced approximately 5 mm apart, was then inserted into the stem. After 3–4 h, the plant was moved to the platform under the objective lens. A gold recording electrode (18 µm in diameter, 15 mm in length, Beijing Doublink Solders Co.,Ltd., Beijing, China) was placed on top of stem. The Ag/AgCl reference electrode was placed in the soil. The operational amplifier and the acquisition system were the same as those described in the Methods.

Supplementary Results

Stable baseline of optical recording for *H. annuus in situ*

When using a 515 nm high pass filter, the intensity of the background autofluorescence (Fb) from

the *H. annuus* stem and petiole of the leaf excited at 488 nm was less than 300 (in arbitrary units, see Supplementary Fig. 1b. This is a 16-bit image with a maximum value of 65535). The time series images show that the variation of the fluorescence intensity (Fv) was approximately 10 (n = 20 samples). This means that the background autofluorescence was stable. After binding of the $DiBAC_4(3)$ dye to the plant, the average fluorescence intensity (F0) was approximately 8000 when excited at 488 nm (Supplementary Fig. 1c). The mean variation of the background fluorescence, expressed as Fv/(F0−Fb)*100%, was 0.13 ± 0.04% (n = 20 samples).

The background autofluorescence had a negligible effect on the optical recording. After small incisions were made in the stem, the plant required at least 2–3 h of rest to reduce the effect from wounding the stem. Before stimulus was applied, a baseline was recorded for 5–10 min to ensure the baseline was stable. When no stimulus was applied, no bioelectrical response was triggered in either the stem or the leaf (Supplementary Fig. 2a). The $\Delta F/F$ changes of the fluorescence were at ± 0.42% (n=20 plant samples, Supplementary Fig. 2b).

Calibration of the fluorescence intensity versus the membrane potential *in vitro*

Calibration of the dye was performed using protoplasts extracted from the *H. annuus* stem. Supplementary Figure 3a shows raw fluorescence images of a protoplast from the *H. annuus* stem. The protoplast responded to different extracellular potassium concentrations (1 mM, 5 mM, 10 mM, 50 mM, 100 mM and 200 mM, pH = 5.7). After subtracting the background noise, the fluorescence intensity was measured. The ratio of K^+-induced fluorescence (Fc) to the initial fluorescence intensity (F0) was recorded using an extracellular potassium concentration of 1 mM. $\Delta F/F$ was then calculated using (Fc-F0)/F0. The fluorescence intensity in the protoplast depended on the extracellular K^+ concentration (Supplementary Fig. 3b). Supplementary Figure 3c shows the fluorescence response using various extracellular K^+ concentrations to determine the relationship between Fc/F0 and the membrane potential. Calibration of the fluorescence versus the change in membrane potential of the protoplast showed that Fc/F0 depended on the membrane potential (Supplementary Fig. 3d). A plot of $\Delta F/F$ versus the membrane potential is shown in Supplementary Figure 3e. Between −118 mV and −100 mV, there was low sensitivity of the fluorescence signal. Between −100 mV and 20 mV, Fc/F0 (or $\Delta F/F$) increased with an increase in the membrane potential. At membrane potential greater than 20 mV, there was no significant change in the fluorescence with an increase in membrane potential.

To determine the cytoplasmic K^+ concentration, the membrane potential of protoplast from stem

with a specific K^+ concentrations (10 mM) in the buffer (Method in manuscript) was recorded using microelectrode. It was 60±10 mV (n=3 protoplasts, mean±S.E.M). As we know, K^+ channels are responsible for most of the plasma membrane conductance, so the cytoplasmic K^+ concentration can be determined according to the Nernst equation as a known extracellular K^+ concentration is given. After calculating, the cytoplasmic K^+ concentration was about 100 mM, which is consistent with the reported value in other papers[4,5].

Calibration using a stem protoplast showed that the fluorescence intensity changed about 1% when the membrane potential was depolarized about 1 mV (Supplementary Fig. 3e). The membrane potential change was induced by a change in the K^+ concentration in the solution.

Spatial and temporal relation between membrane potential and fluorescence

We observed differences between the simultaneous traces shown in Figure 2 and those in supplementary Figure 5b. Extracellular recording using Ag/AgCl electrodes reflects the superposition of the AP signals from the cells around the electrode at a site. The action potentials that are recorded extracellularly differ from those recorded intracellularly. The shape of the waveform for any single extracellular action potential depends on the exact geometry of its contact with the electrode and its position. We used extracellular recording to monitor the occurrence of the action potential or to record the activity of an entire population of cells. In addition, the APs in Figure 2 are taken from a very small selected range that includes smaller numbers of cells, while the extracellular APs represent the activity of an entire population of cells in contact with the Ag/AgCl electrode.

The challenges of making optical recordings of plants *in situ* are shown in Figures 2 and 3. Multi-layered cells are present in in situ recordings, and each of the cells has a different focal plane. Unlike single cell or monolayer cells, the stem of plant in situ represents a thick sample for optical recording. Thus, the $\Delta F/F$ in a selected range reflects the signals from several cells in overlapping layers and is dependent on position. The $\Delta F/F$ profiles reflect the potential changes of all cells that can generate fluorescence in the analysis region, and in this scenario, the results will be rather more complex than those of a single cell recording. The profiles of regions 1, 2, 3, and 4 in Figure 2 are different; however, uniform waveform profiles also exist, because the profiles in regions I, II, III, and IV of Figure 3 remain synchronous with the extracellular APs shown in Supplementary Figure 5d–f. The same results were found in Supplementary Figure 6 and Supplementary Figure 7 . Thus, although there are differences between the $\Delta F/F$ profiles themselves, or between the $\Delta F/F$ profiles and the

extracellular recordings of the APs, the optical recording can map these differences in a manner that traditional electrophysiological methods cannot.

While the method presented here can map the distribution of the plant electrical activity with high spatial resolution, the analysis method used accurately links the spatial and temporal relationships between the membrane potential and the fluorescence from the overlapping layers of cells， and is still worthy of in-depth study in the future.

Measurement of propagation velocity using both extracellular recording and optical recording

The velocity of electrical stimulation-induced extracellular AP measured by the traditional recording method was 1.64±0.12 mm/s (n=3 plants, mean ± S.E.M.). The results are shown in Supplementary Figure 17 and Supplementary Table 2. Although the observed area was approximately 3.0 mm ×0.15 mm, we were able to calculate the velocity of propagation with a 4× objective at a 5 Hz sampling rate using optical recording. The velocity measured by the optical recording method was 1.75±0.14mm/s (n=3 plants, mean ± S.E.M.). The propagation speeds of extracellular AP were also similar to those that were reported in in the literature[6-9].

Next, we obtained optical recordings of variation potential stimulated by heat with a 2× objective at a 1.2 Hz sampling rate, and then calculated the velocity. The results are shown in Supplementary Figure 18 and Supplementary Table 2. There was no significant difference between the two methods for velocity measurement ($p < 0.05$), and results agree with those that have been reported in the literature[6,10]. However, the signal-to-noise ratio (SNR) was reduced when using the 2× objective. The accuracy of the velocity measured by the optical recording method was limited by the speed and sensitivity of our CCD. However, a high performance CCD may circumvent this problem and improve measurement accuracy.

Supplementary Note

Note 1

In the early stages of this study, we tried to perform non-invasive optical recording using a confocal instrument (Leica Sp5), but found that it was difficult to obtain a clear bright field or fluorescence image (Supplementary Fig.19) in the stem because its rough surface caused increased scattering that tended to blur the focus for thicknesses from 2 mm to 2.5 mm, which appeared to be too large for the confocal instrument. Many previous reports have indicated that an excision is necessary to

ensure that the cells are well stained with dye, even in a leaf vein, when using confocal instruments[11,12]. To make $DiBAC_4(3)$ bind well to the membranes, we prepared specimens by the method described in the manuscript (Methods in manuscript).

Note 2

In addition, we recorded the oxygen fluxes of exposed tissue of intact plant in the bath solution each hour during the dye loading process. The O_2 fluxes measurements in our experiments were performed as previously reported[13]. Supplementary Table 3 shows that the value of oxygen influx exposed tissue is approximately 9.68 ± 1.51 pmol•cm^{-2}•s^{-1} (n= 5 plants, mean $\pm$ S.D.).Thus, in our study the oxygen consumption of partial submerged stem within four hours was approximately $3.16 \pm 0.56 \mu g$ (n= 5 plants, mean $\pm$ S.D.), which is very small to total oxygen content in the bath solution.

We also performed an aerated control experiment, which showed that there were no differences in fluorescence intensity between the control group and the aerated group (see Supplementary Figure 20). Thus, these data suggest that partial immersion in water solution within a limited time (approximately 4h) does not affect our recordings.

Note 3

Please visit website (https://mega.nz/#F!hZhi2Koa!fIX4Fxslcph-ynb4lL1WaA). There is a compressed files, 'Supplementary software and User Guide.zip'. In the file, there are test data (114 pictures in 'data' directory), software source files and user guide file. After download and unzip the compressed file, please read the "User Guide of Supplementary software.pdf" before analysis using supplementary software.

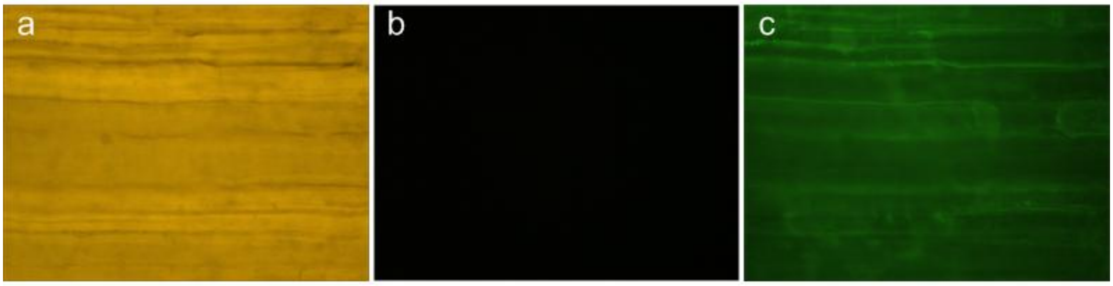

Figure S1 | Background autofluorescence compared with average intensity after binding $DiBAC_4(3)$ using optical recording. (a) Bright field view of the sunflower stem section. (b) Autofluorescence from the surface of the exposed tissue of stem without the dye when excited at 488 nm, where the intensity was less than 300 (for a 16-bit image, the maximum intensity value was 65535). The variation of the intensity was approximately 10 (n = 20 plant samples) as determined from the time lapse images. (c) An image of the fluorescence distribution from the cut section with 10 μM $DiBAC_4(3)$. The average intensity excited at 488 nm, was about 8000 (n = 20 plant samples). The variation in the autofluorescence had a negligible effect on the recording using $DiBAC_4(3)$.

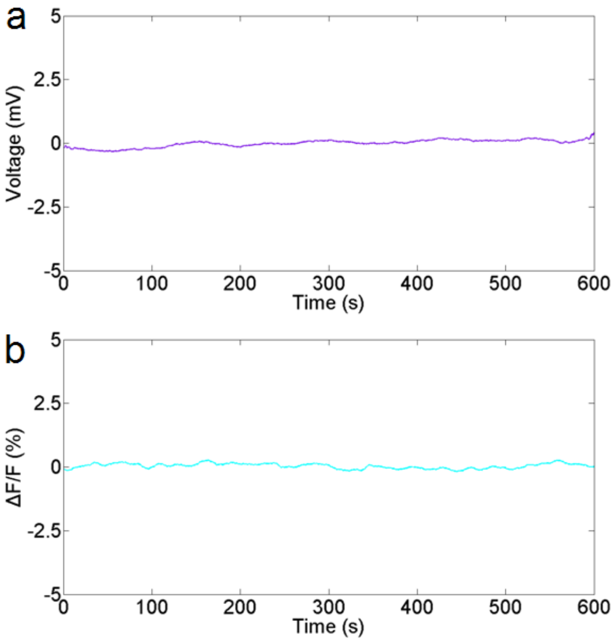

Figure S2 | Baselines of the electrical and optical recordings. (a) The change of the potential recorded using the Ag/AgCl electrode. When there was no electrical stimulus, no AP was triggered. (b) The baseline of the optical recording. If there was no AP in the *H. annuus* stem, the fluorescence intensity change was only affected by photobleaching. The ΔF/F baseline was then at a low level compared with the dye response.

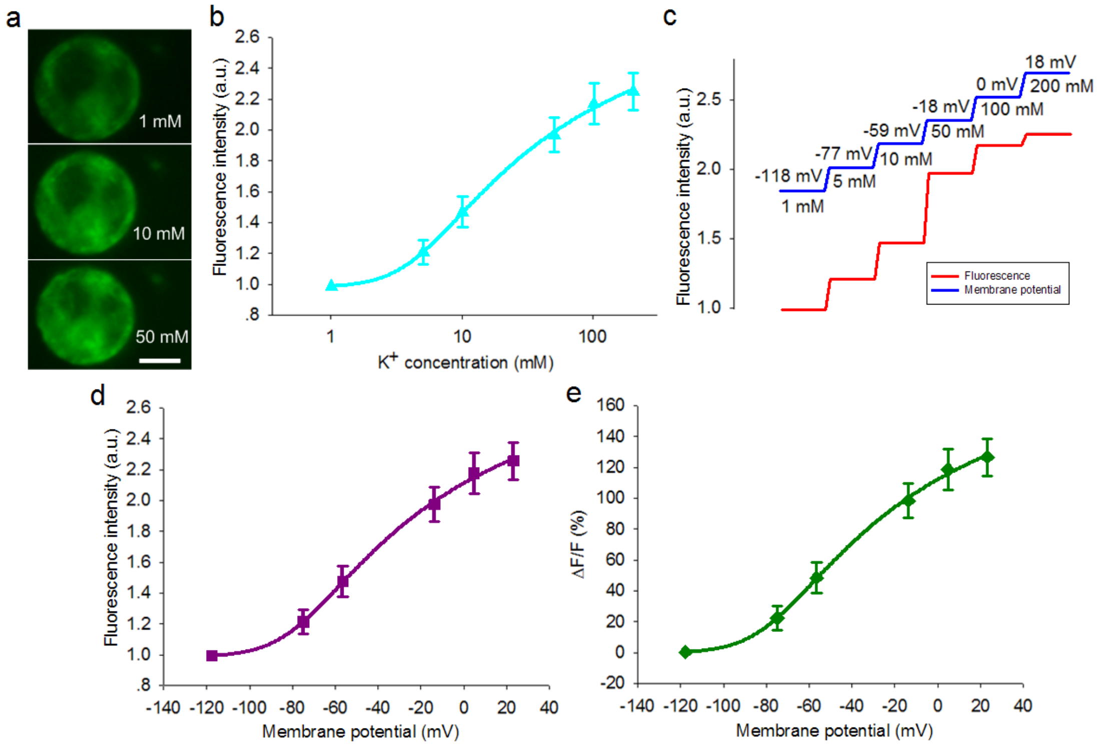

Figure S3| Dye calibration performed using protoplasts extracted from the *H. annuus* stem. (a) Raw fluorescence images showing the response of the protoplasts extracted from *H. annuus* to different K^+ concentrations. (b) Relative fluorescence Fc/F intensity at different K^+ concentrations (n = 10 protoplasts, mean ± S.E.M.). (c) The dependence of the fluorescence intensity on the membrane potential induced by different K^+ concentrations. The upper trace indicates the membrane potential and the corresponding K^+ concentrations (*n* = 10 protoplasts, mean ± S.E.M.). The lower trace is the relative fluorescence induced by different K^+ concentrations. The membrane potentials were obtained according to the Nernst equation. (d) A plot of the fluorescence intensity, Fc/F, versus the K^+-induced membrane potential. There is a relationship between the membrane potential and the normalized fluorescence (*n* = 10 protoplasts, mean ± S.E.M.). (e) The dependence of the fluorescence, ΔF/F, on the membrane potential. The relationship was derived from (d).

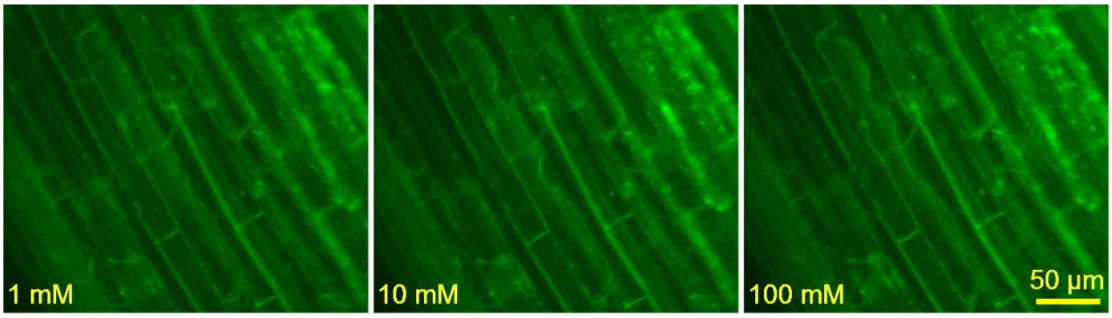

Figure S4 | Raw fluorescence images showing the response of the *H. annuus* stem to different K^+ concentrations *in situ*.

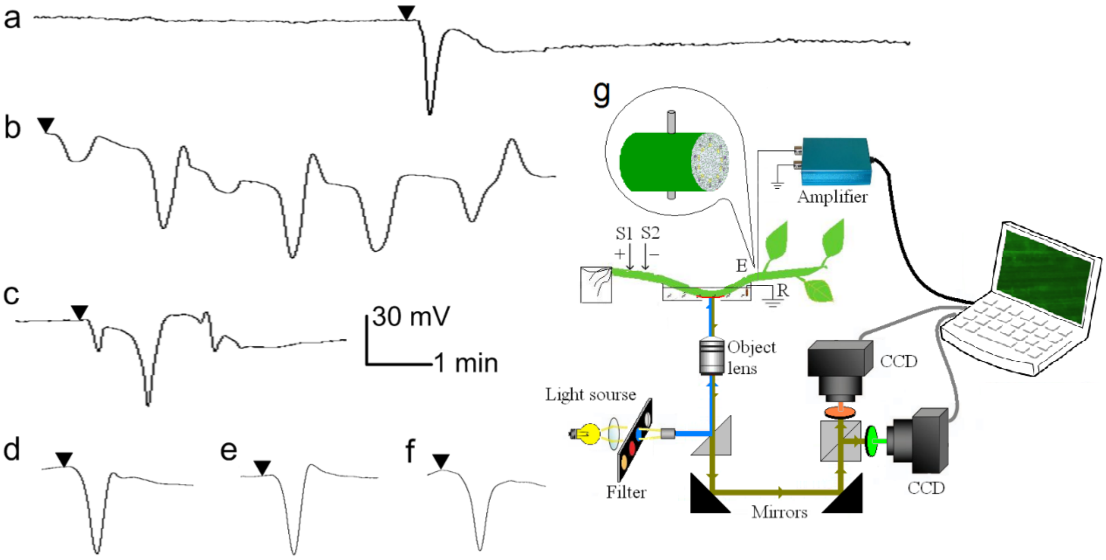

Figure S5 | APs recorded using an Ag/AgCl electrode in the *H. annuus* stem. (a) A 5 min Ag/AgCl electrode recording. This recording was before the stimulus and indicates that the stem potential was stable after *H. annuus* was rested for several hours. After the stimulus, an AP was induced. (b)–(c) A recorded series of APs and (d)–(f) recorded single AP. (g) A Ag/AgCl electrode inserted across the stem. The arrow indicates the timing of the electrical stimulation in a-f.

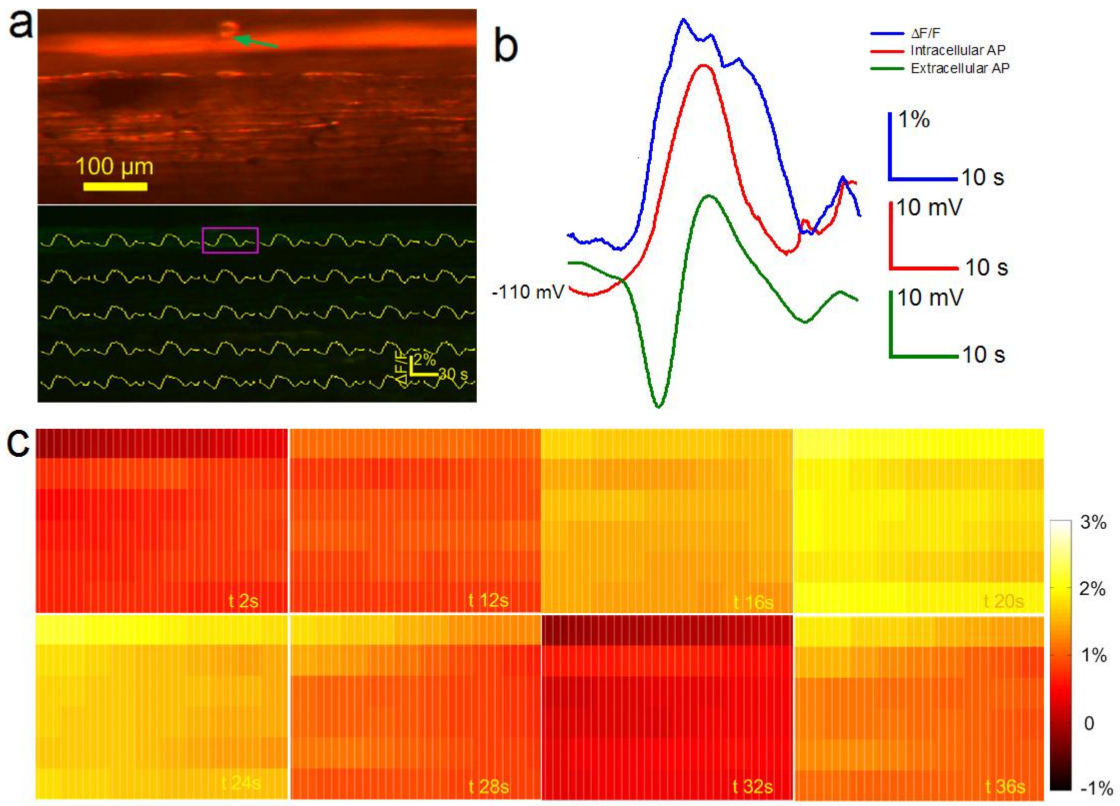

Figure S6 | Simultaneous recordings of intracellular AP using a glass microelectrode(tip diameter smaller than 0.5μm)，extracellular AP using an Ag/AgCl electrode and the DiBAC$_4$(3) fluorescence using an upright fluorescence microscope to map the induced bioelectrical signal of stem by electrical stimulation. (a) Bright field and fluorescence images of the *H. annuus* stem. The location of the glass microelectrode for intracellular recording is indicated by the green arrow in the bright field image. ΔF/F distribution curves are displayed in a raw fluorescence image. (b) Overlay of the mean optically and electrically recorded both intracellular AP and extracellular AP waveforms. (c) Time lapse ΔF/F pseudocolor images, indicating the variation of the fluorescence intensity.

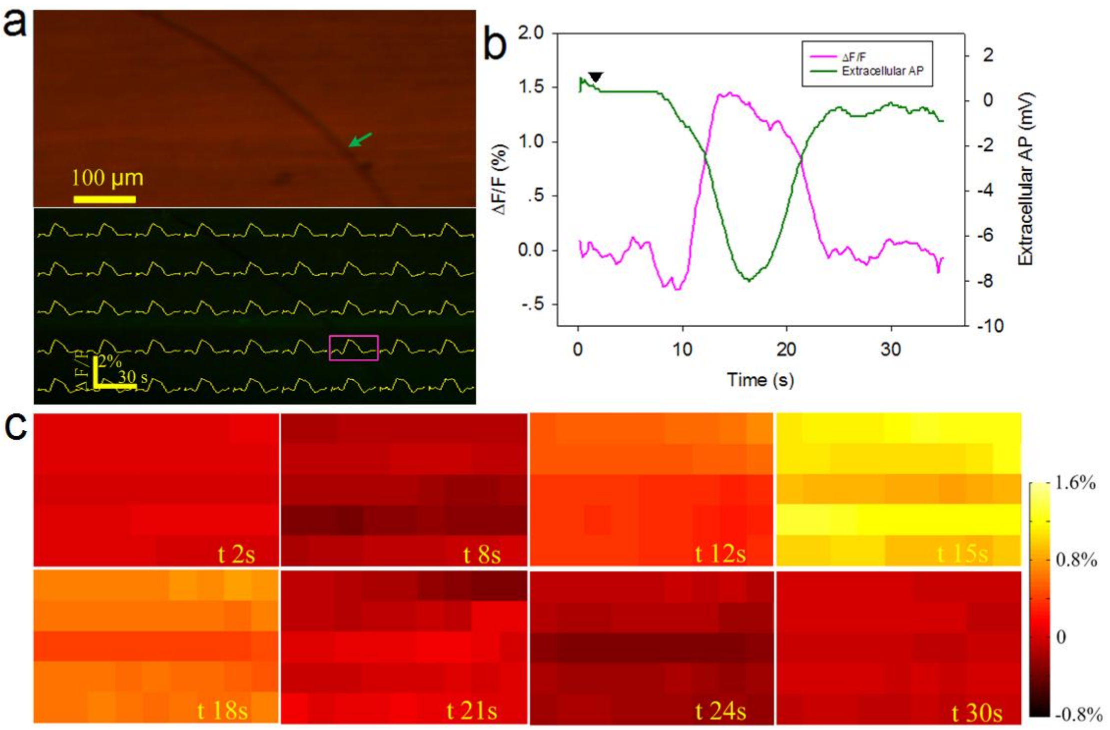

Figure S7 | Simultaneous recordings of extracellular APs using a gold electrode(18 μm in diameter, 15 mm in length) and the DiBAC$_4$(3) fluorescence using an upright fluorescence microscope to map the induced bioelectrical signal of stem by electrical stimulation (a) Bright field and fluorescence images of the *H. annuus* stem. The location of the gold electrode for extracellular recording is indicated by the green arrow in the bright field image. ΔF/F distribution curves are displayed in a raw fluorescence image. (b) Overlay of the mean optically and electrically recorded action potential waveforms. (c) Time lapse ΔF/F pseudocolor images, indicating the variation of the fluorescence intensity.

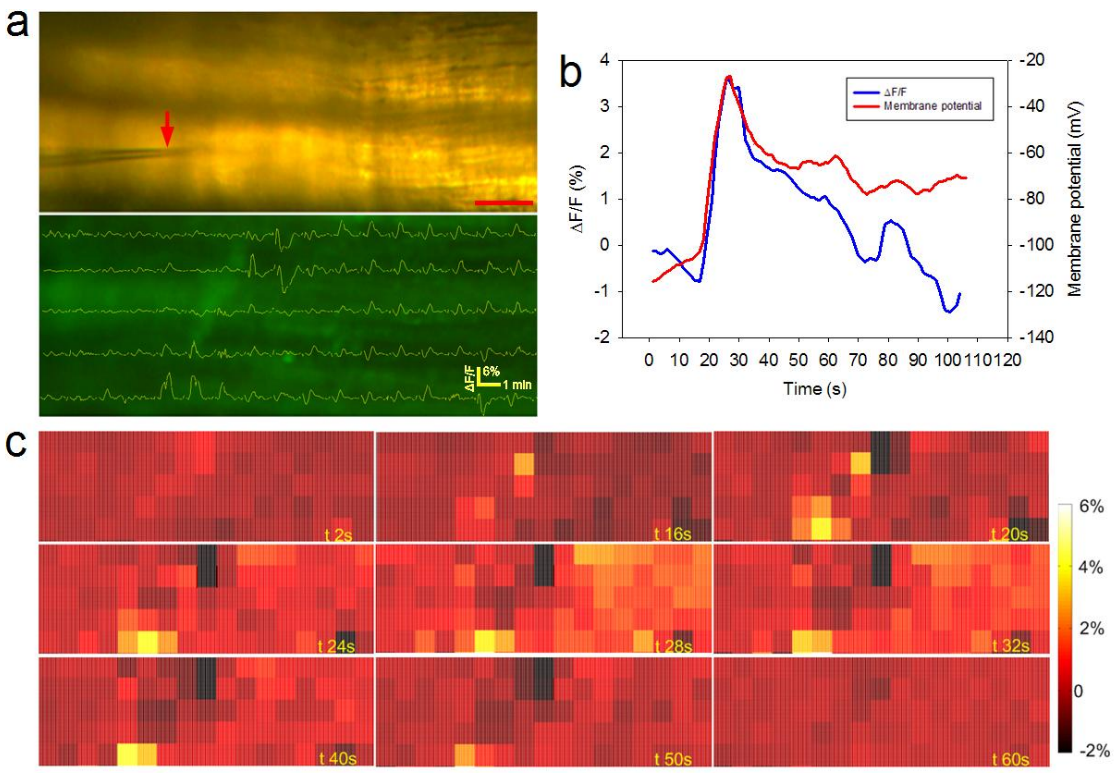

Figure S8 | Simultaneous recordings of electrical activity using a microelectrode and the DiBAC$_4$(3) fluorescence using an upright fluorescence microscope to map the bioelectrical signal of a leaf stimulated by heat. (a) Bright field and fluorescence images of the *H. annuus* leaf petiole. The location of the microelectrode for intracellular recording is indicated by the red arrow in the bright field image. ΔF/F distribution curves are displayed in a raw fluorescence image. (b) Overlay of the mean optically and electrically recorded variation potential waveforms. (c) Time lapse ΔF/F pseudocolor images, indicating the variation of the fluorescence intensity.

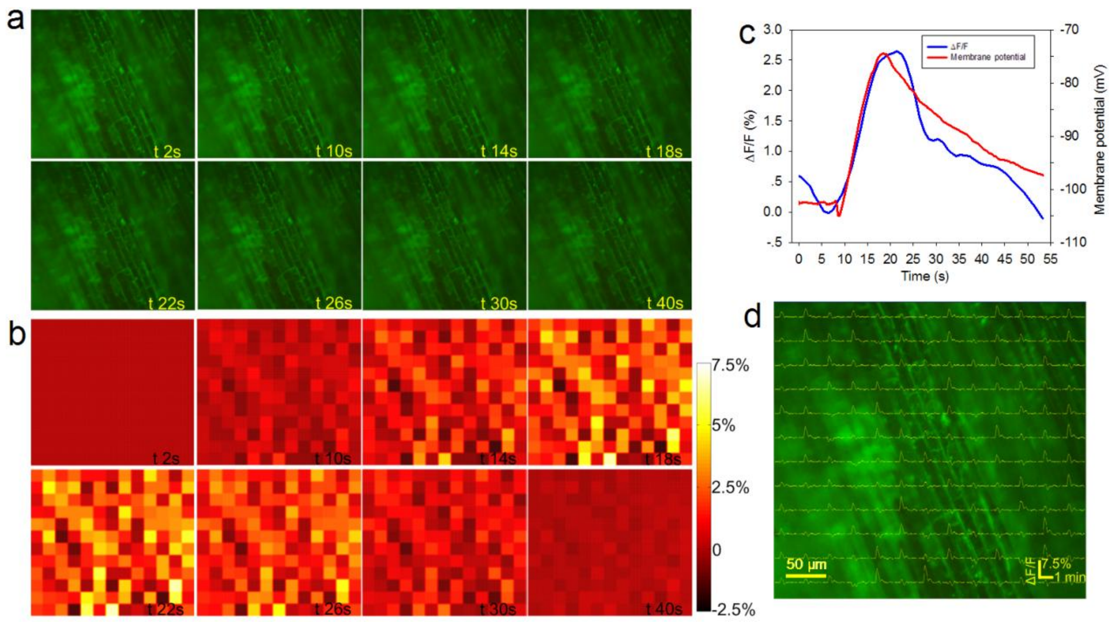

Figure S9 | Typical optical recording enabled the mapping of VPs *in situ* in the stem of *H. annuus*. (a) A series of consecutive raw fluorescence images from a typical recording. (b) Time lapse ΔF/F pseudocolor images derived from (a), where the variation in the fluorescence intensity can be clearly observed. (c) Overlay of the mean optically and electrically recorded variation potential waveforms. (d) The ΔF/F curves were mapped on a raw fluorescence image. Each curve represents the change in the fluorescence intensity of the region around it. The maximum amplitude of the ΔF/F signal in the recording region is approximately 7.5 % (n=5 recording repeats, 2 plant samples).

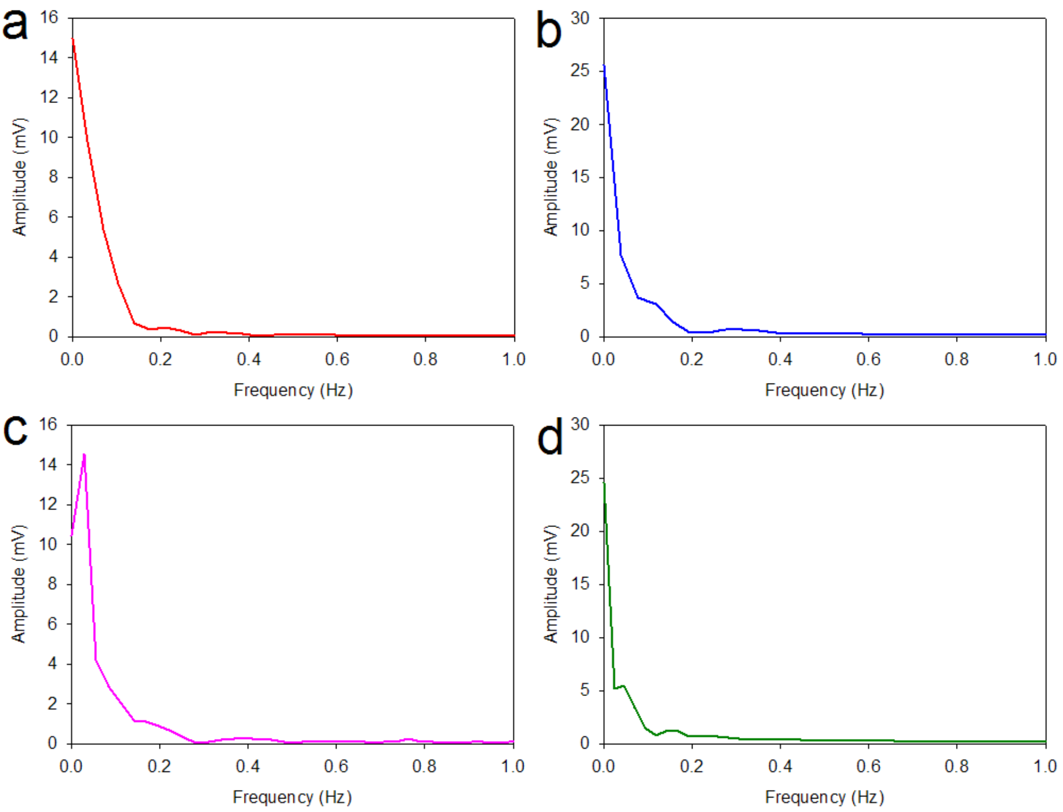

Figure S10 | The frequency spectrum of action potentials from Supplementary Figure 5b,d-f, showing that the frequency domain of electrical activity was lower than 0.3 Hz.(a) The frequency spectrum of AP in Figure 5b. (b) The frequency spectrum of AP in Figure 5d. (c) The frequency spectrum of AP in Figure 5e. (d) The frequency spectrum of AP in Figure 5f.

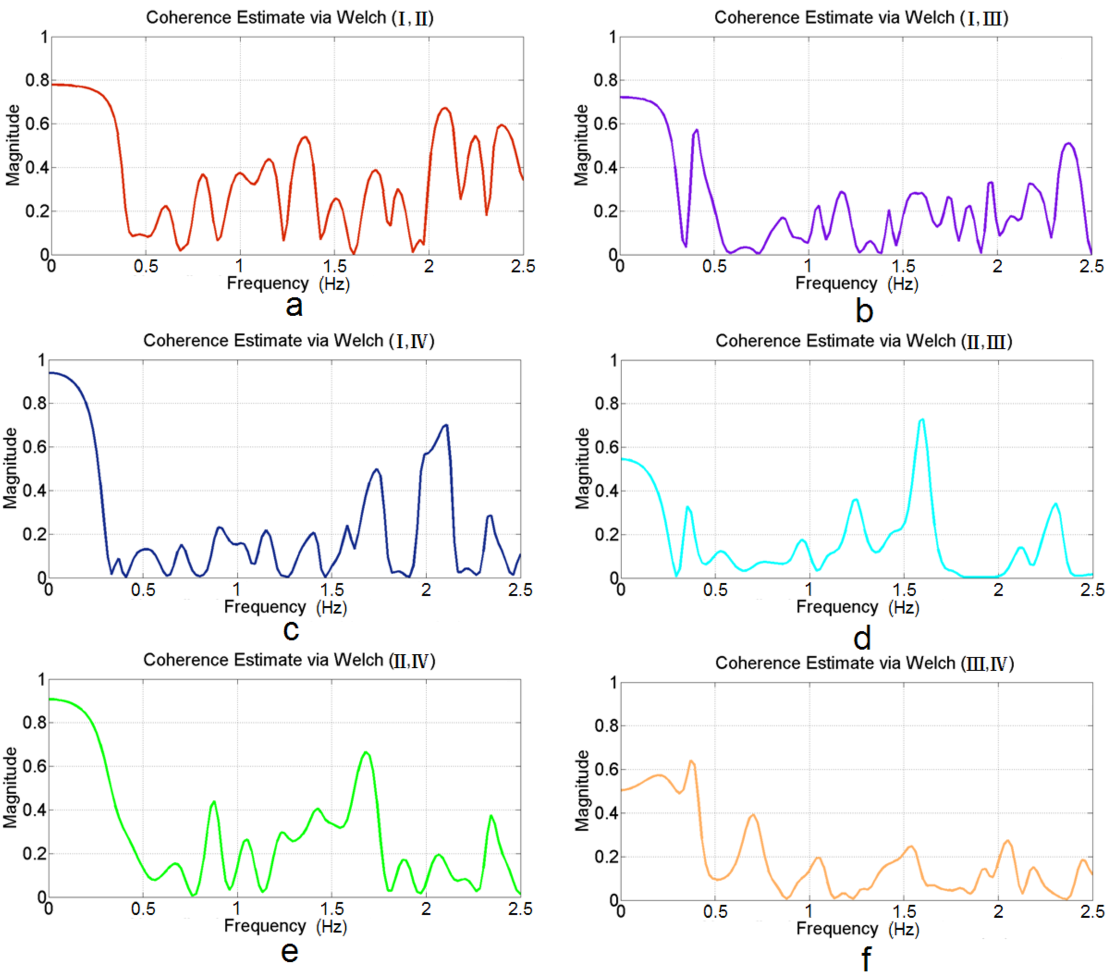

Figure S11 | Coherence analysis for the different signals from the numbered adjacent regions showed in Figure 3a. (a) Frequency of coherence between the numbered regions I and II. (b) Frequency of coherence between the numbered regions I and III. (c) Frequency of coherence between the numbered regions I and IV. (d) Frequency of coherence between the numbered regions II and III. (e) Frequency of coherence between the numbered regions II and IV. (f) Frequency of coherence between the numbered region III and IV. The maximum coherence magnitude (Coh_{max}) of the signals which is nearly or higher than 0.8 can be found in a, b, e and f. Also, a Coh_{max} that is nearly 0.6 can be found in c, d. In all samples, the Coh_{max} appears in the low frequency range. Coherence analysis showed that there are high correlations ($Coh_{max} > 0.5$, $P < 0.005$, *t*-test) in the frequency domain between the different signal regions.

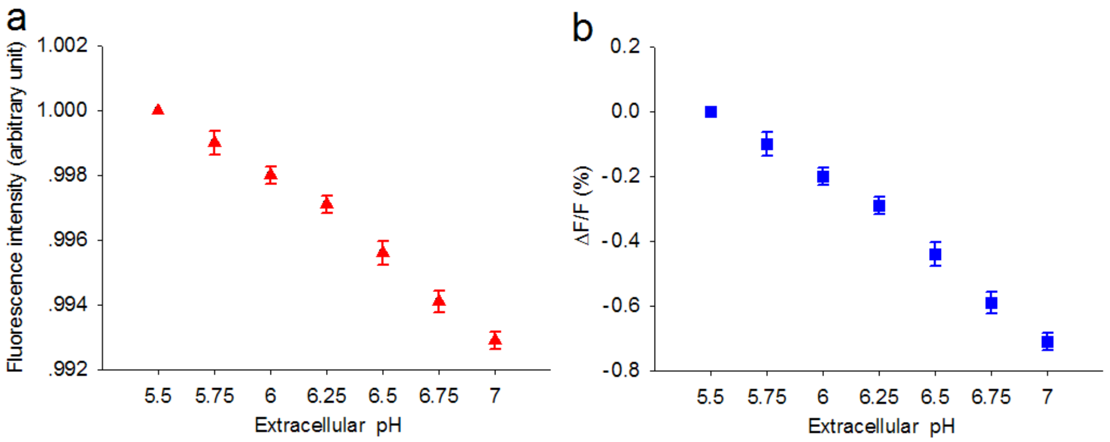

Figure S12| Effect of bath solutions with different pH levels on the fluorescence intensity *in situ* in the *H. annuus* stem and leaf (n = 8 plant samples, mean ± S.E.M.). When the pH of the solution changed, the fluorescence intensity had a slight variation (maximum of about 0.68%±0.05%). When compared with the AP-induced or VP-induced change in ΔF/F of 5–8%, this was negligible. (a) The relative fluorescence intensity versus the extracellular pH. (b) The relationship between ΔF/F and the extracellular pH derived from (a).

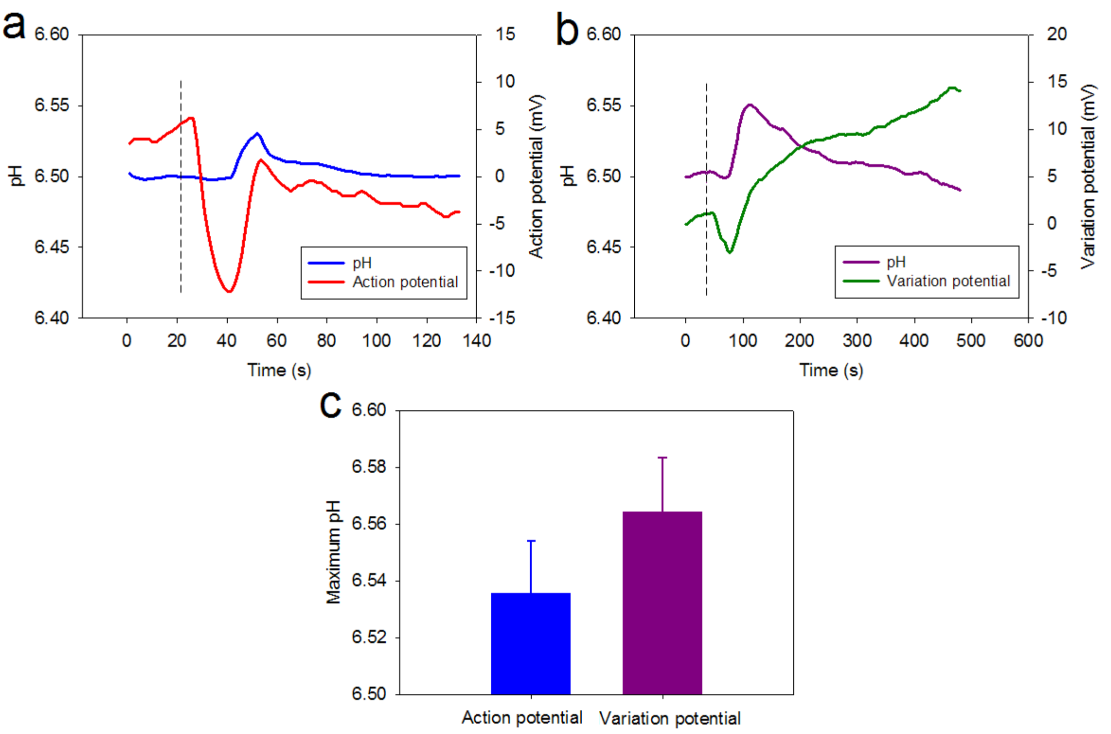

Figure S13| Simultaneous recording the extracellular pH change at approximately 5μm close to the tissue surface of the stem and electrical activity. (a) A small pH change was observed after action potential was induced by electrical stimulation applied to the stem. The dash line indicates the

stimulation point. (b) There was also a small pH change after variation potential was induced by heat stimulation applied to the leaf. The dash line indicates the stimulation point. (c) In the repeated experiments, the maximum pH induced by action potential is about 6.55, and the pH change is smaller than 0.06 (n=4 plant samples， mean ± S.E.M.). For variation potential, the maximum pH is about 6.58, and the pH change is smaller than 0.1 (n=5 plant samples， mean ± S.E.M.)

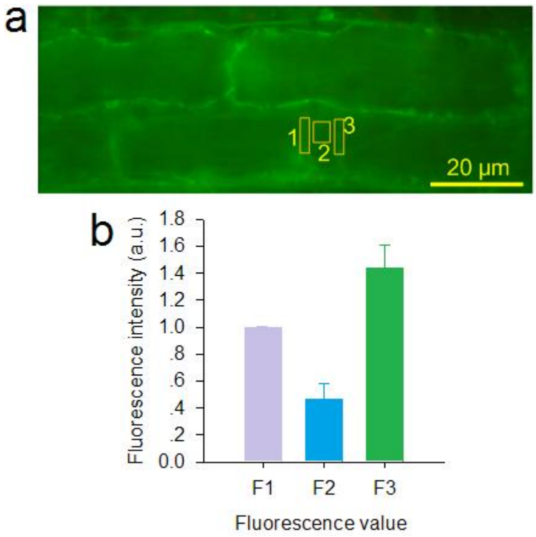

Figure S14 | Plasmolysis indicated that there are some components of the fluorescence that contribute to the signal and others that do contribute to the resting fluorescence. Shown here are fluorescence images from a slice of *H. annuus* stem labeled with DiBAC$_4$(3) and excited at 488 nm after plasmolysis using a fluorescence microscope. Plasmolysis of the cell in the cortex of the *H. annuus* stem indicated that there was higher fluorescence in the cell wall after it was stained with the DiBAC$_4$(3) dye. The yellow rectangles (approximately 300 pixels in size) show the positions numbered 1, 2 and 3, where the normalized fluorescence F1, F2 and F3 were analyzed, respectively. (a) A typical fluorescence image after plasmolysis using a fluorescence microscope. (b) The averaged values of F1, F2 and F3 (n = 4 cells in total from three separate experiments, mean ± S.E.M.).

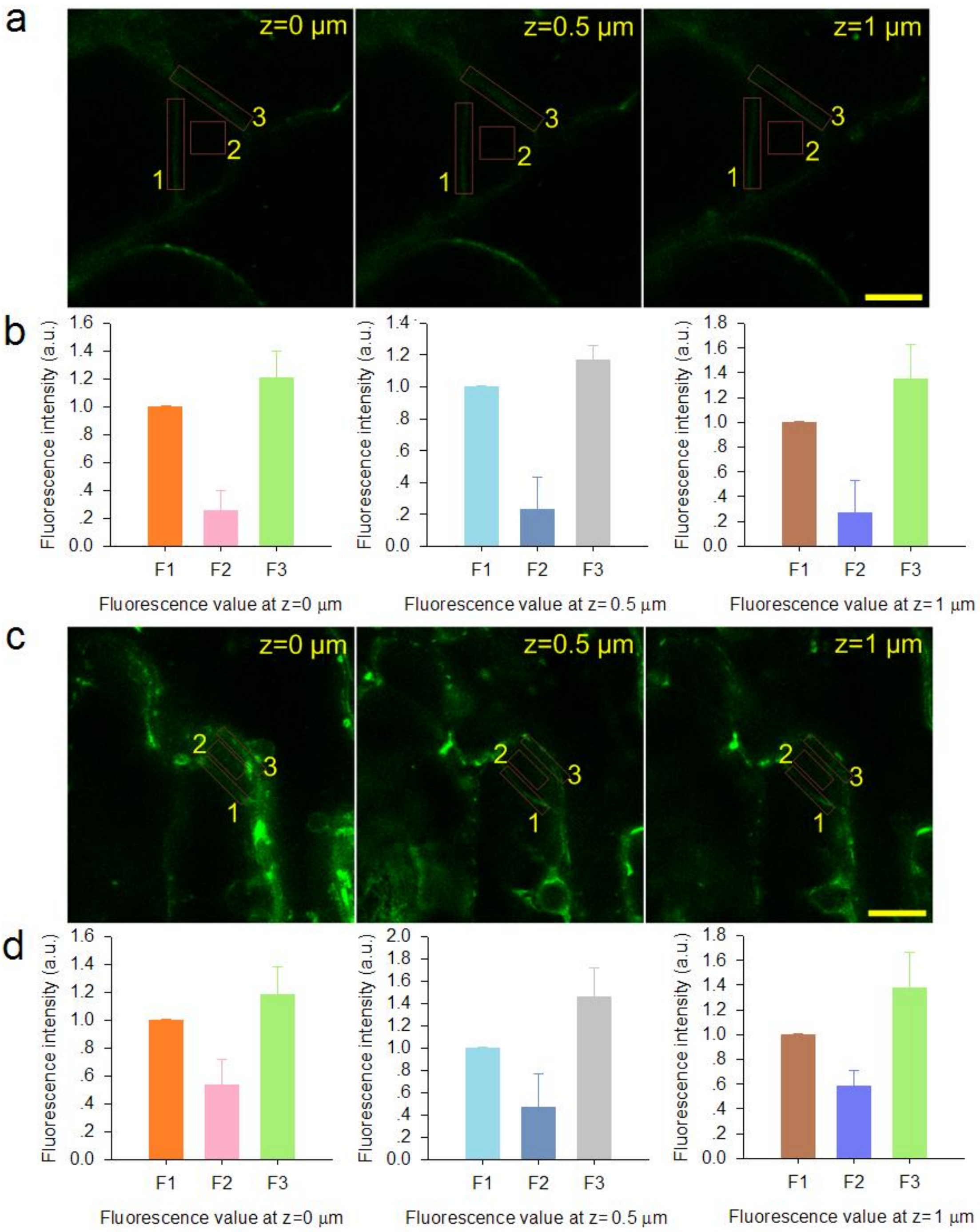

Figure S15 | Confocal laser scanning microscopy (CLSM) fluorescence images of a slice from the stem and leaf of *H. annuus* labeled with DiBAC$_4$(3) and excited at 488 nm after plasmolysis. The dark red rectangles (approximately 900 pixels in size) show the positions numbered 1, 2 and 3, where the fluorescence intensities F1, F2 and F3 were analyzed, respectively. (a) Typical CLSM images (in the XY plane) of a slice from the stem at Z = 0 μm (left), Z = 0.5 μm (middle), and Z = 1.0 μm (right). The bar is 20 μm. (b) The average values of F1, F2 and F3 along the Z axis at Z = 0 μm (left), Z = 0.5 μm (middle), and Z = 1.0 μm (right), respectively. These values were derived from (a), where n = 6 cells in

total from four separate experiments, mean±S.E.M. (c) Typical CLSM images (XY plane) of a slice from a leaf at Z = 0 μm (left), Z = 0.5 μm (middle) and Z = 1.0 μm (right). The bar is 20 μm. (d) The average values of F1, F2 and F3 along the Z axis where Z = 0 μm (left), Z = 0.5 μm (middle) and Z = 1.0 μm (right), respectively. These values were derived from (c), where n = 6 cells in total from four separate experiments, mean ± S.E.M.

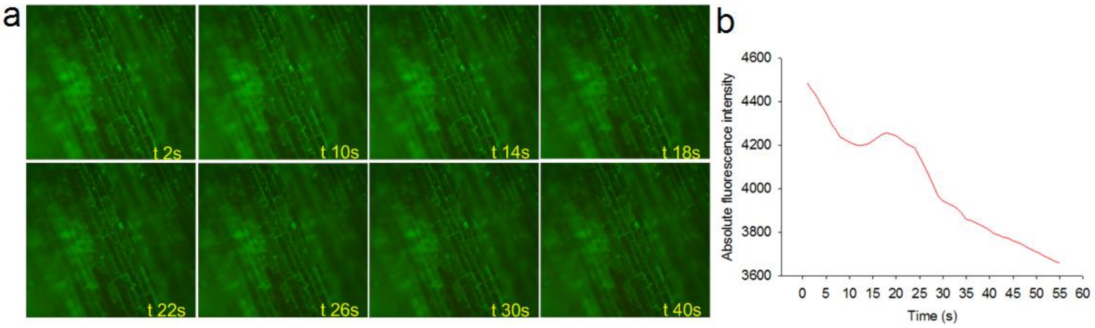

Figure S16 | Raw optical signal of the fluorescence images of the *H. annuus* stem without correcting for photobleaching. (a) A series of consecutive raw fluorescence images induced by electrical stimulation. (b) The raw traces from fluorescence images without correcting for photobleaching.

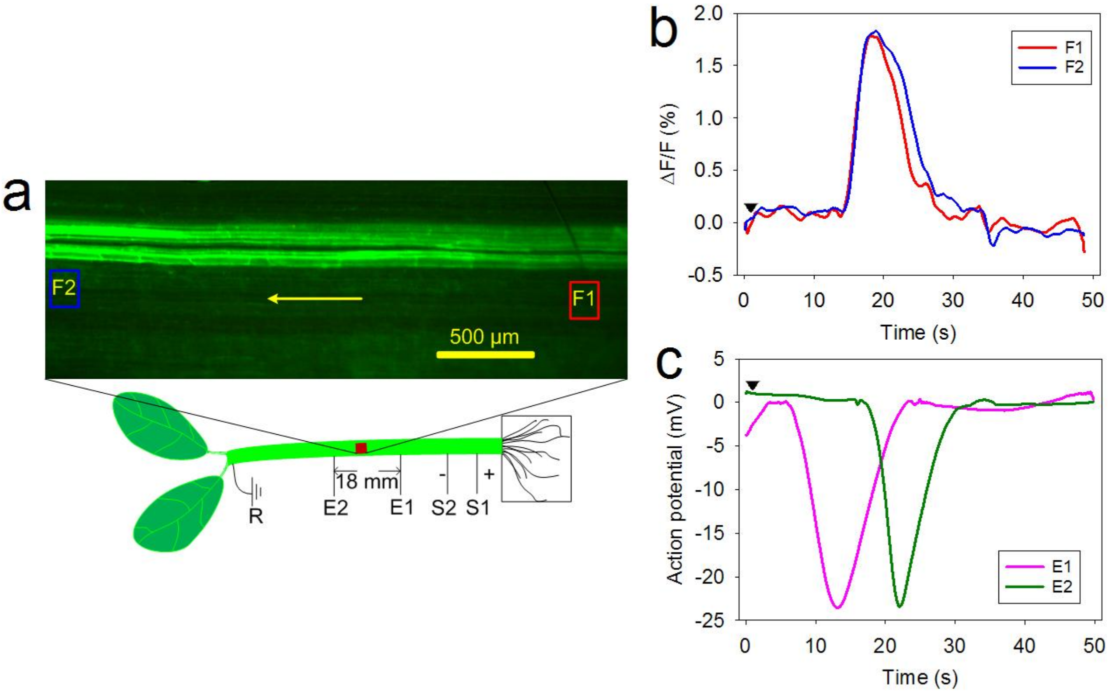

Figure S17 | Propagation velocity of APs from both extracellular recording and optical recording. (a) Schematic illustrating experimental approach for both extracellular recording and optical recording. Proximal of propagation (red rectangle) and distal of propagation (blue rectangle) shown in a

fluorescence image by optical recording, with the area of analysis marked by the rectangle. S1 and S2 are the stimulating electrodes using 0.1 mm Pt wires. S1 is the anode, E1 and E2 is the Ag/AgCl electrodes, and R is the reference electrode connected to the ground. (b)The two curves obtained from proximal (red) and distal (blue) by optical recording; (c) The two curves obtained from E1 and E2 by extracellular recording. The arrow represents the direction of signal propagation. The triangle indicates the timing of the stimulation.

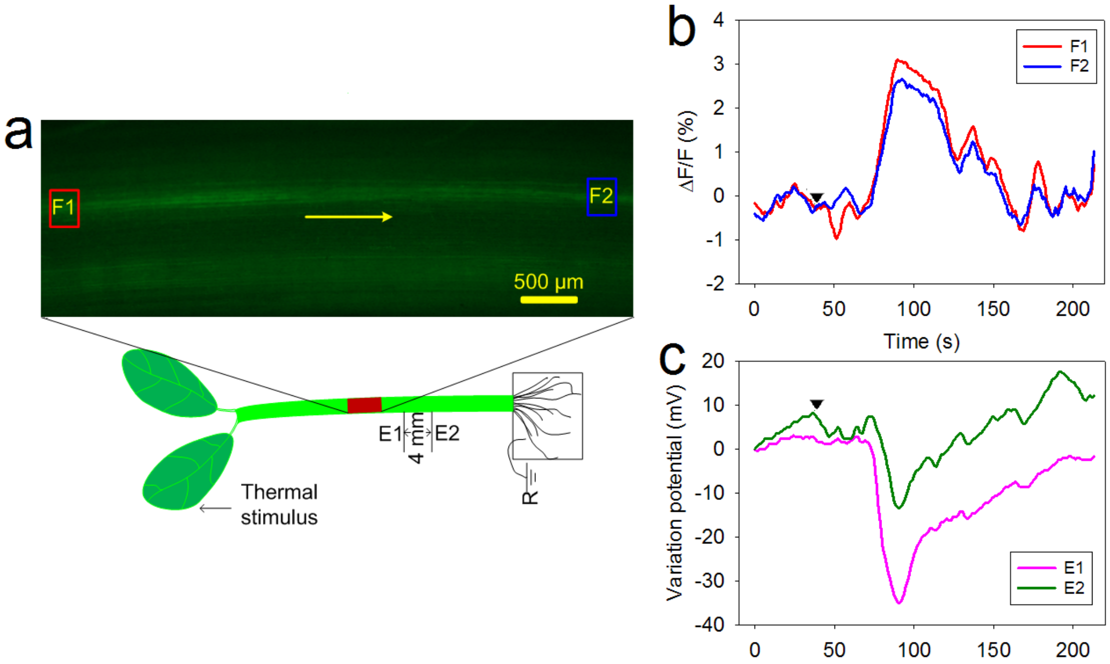

Figure S18| Propagation velocity of heat-induced VPs from both extracellular recording and optical recording. (a) Proximal of propagation (red square) and distal of propagation (blue square). (b) Two curves obtained from proximal (blue) and distal (line) by optical recording. (c) The two curves obtained from proximal (E1) and distal (E2) by extracellular recording. The arrow represents the direction of signal propagation. The triangle indicates the timing of the stimulation.

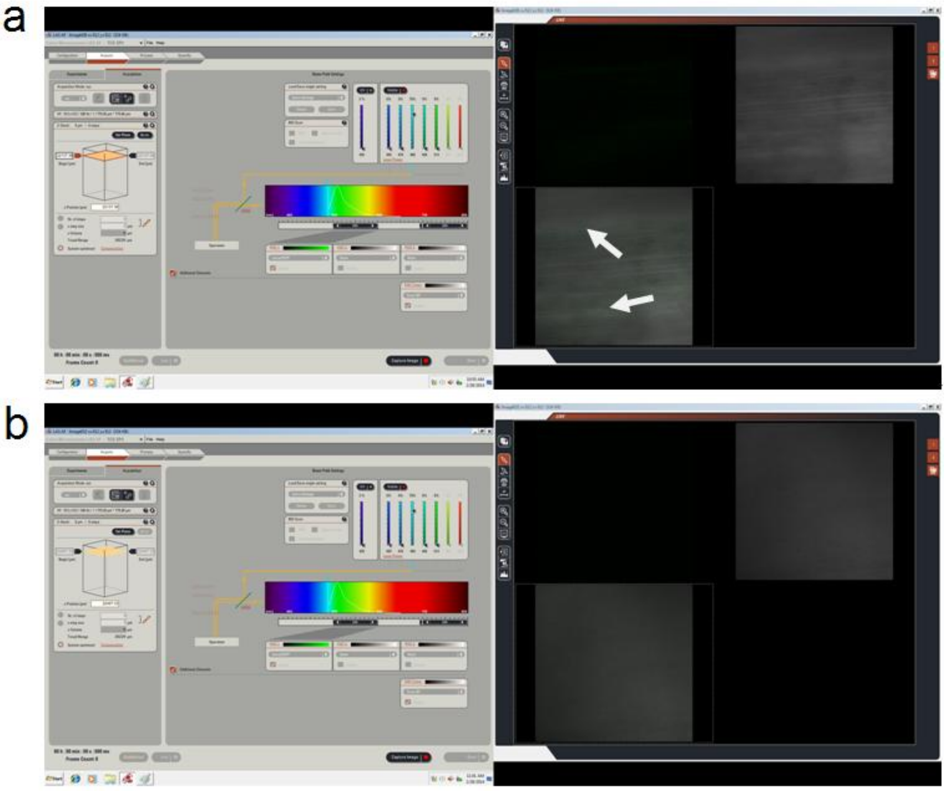

Figure S19 Non-invasive optical recording using confocal instrument in the *H. annuus* stem. Without cutting, it was difficult for the dye to bind to or enter cells in the stem. (a) Only weak fluorescence (indicated by the white arrows) was detected in the stem surface. (b) No fluorescence was recorded at a 300 μm depth in the stem.

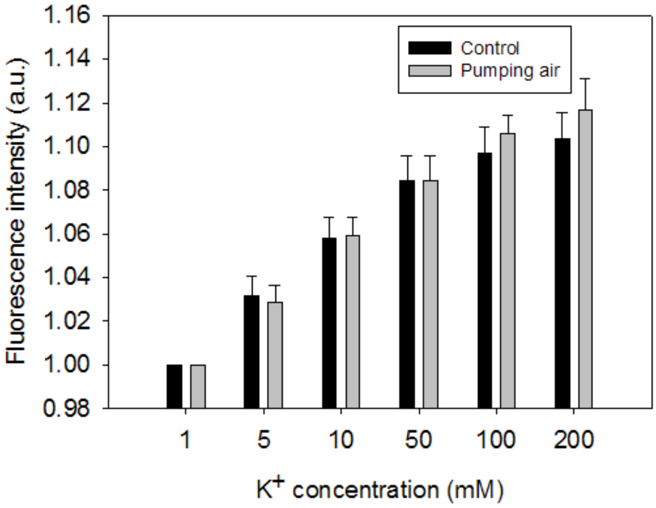

Figure S20 | Comparison of fluorescence intensity of the exposed phloem tissue stained with DiBAC4(3) at different K^+ concentration, after loading with $DiBAC_4(3)$ in a buffer for 230–250 min at room temperature. There are no differences in fluorescence intensity between the control group and the aerated group (n=5 plants, $p < 0.05$, student *t-test*)

Supplementary Table 1

Calibration solutions

Solution name	Solution composition
Solution 1	0.5 mM $CaCl_2$, 10 mM sucrose, 1.5 mM KCl, 2.5 mM HEPES-NaOH adjusted pH to 7.0
Solution 2	0.5 mM $CaCl_2$, 10 mM sucrose, 1.5 mM KCl, 2.5 mM HEPES-NaOH adjusted pH to 6.5
Solution 3	0.5 mM $CaCl_2$, 10 mM sucrose, 1.5 mM KCl, 2.5 mM HEPES-NaOH adjusted pH to 6.0

Supplementary Table 2

Propagation velocity from both extracellular recording and optical recording (n=3 plants，mean ± S.E.M.)

	Propagation velocity (mm/s)	
	Extracellular recording	Optical recording
AP	1.64±0.12	1.75±0.14
VP	1.48±0.11	1.55±0.12

Supplementary Table 3

Oxygen influxes of partial submerged stem within four hours during the dye loading process (n= 5 plants, mean ± S.E.M.)

Time (h)	Oxygen influxes (pmol•cm^{-2}•s^{-1})
1	11.83±6.39
2	9.64±1.99
3	8.66±1.92
4	8.59±1.22

Supplementary references

1 Shabala, S. & Newman, I. Light-induced changes in hydrogen, calcium, potassium, and chloride ion fluxes and concentrations from the mesophyll and epidermal tissues of bean leaves. Understanding the ionic basis of light-induced bioelectrogenesis. *Plant Physiol*. **119**, 1115-1124 (1999).

2 Felle, H. H., Kondorosi, E., Kondorosi, A. & Schultze, M. The role of ion fluxes in Nod factor signalling in Medicago sativa. *Plant J*. **13**, 455-463 (1998).

3 Percey, W. J. et al. Ion transport in broad bean leaf mesophyll under saline conditions. *Planta* **240**, 729-743 (2014).

4 Clint, G. M. & Blatt, M. R. Mechanisms of fusicoccin action: evidence for concerted modulations of secondary K^+ transport in a higher plant cell. *Planta* **178**, 495-508 (1989).

5 Leigh, R. A. Potassium homeostasis and membrane transport. J. *Plant Nutr. Soil Sc*. **164**, 193-198 (2001).

6 Stankovic, B., Witters, D. L., Zawadzki, T. & Davies, E. Action potentials and variation potentials in sunflower: An analysis of their relationships and distinguishing characteristics. *Physiol. Plantarum* **103**, 51-58 (1998).

7 Zawadzki, T., Davies, E., Dziubinska, H. & Trebacz, K. Characteristics of Action-Potentials in *Helianthus- Annuus. Physiol Plantarum* **83**, 601-604 (1991).

8 Zawadzki, T., Dziubińska, H. & Davies, E. Characteristics of action potentials generated spontaneously in Helianthus annuus. *Physiol Plantarum* **93**, 291-297 (1995).

9 Stahlberg, R., Stephens, N. R., Cleland, R. E. & Van Volkenburgh, E. Shade-Induced Action Potentials in Helianthus annuus L. Originate Primarily from the Epicotyl. *Plant. Signal .Behav* .**1**, 15-22 (2006).

10 *Dziubinska*, H., Trebacz, K. & Zawadzki, T. Transmission route for action potentials and variation potentials in *Helianthus annuus* L. *J. Plant. Physiol.* **158**, 1167-1172 (2001).

11 Wang, N. & Fisher, D. B. The use of fluorescent tracers to characterize the post-phloem transport pathway in maternal tissues of developing wheat grains. *Plant Physiol*. **104,** 17-27 (1994).

12 Furch, A. C. U., Zimmermann, M. R., Will, T., Hafke, J. B. & van Bel, A. J. E. Remote-controlled stop of phloem mass flow by biphasic occlusion in Cucurbita maxima. *J. Exp. Bot*. **61**, 3697-3708 (2010).

13 Zeng, F. R. et al. Linking oxygen availability with membrane potential maintenance and K^+ retention of barley roots: implications for waterlogging stress tolerance. *Plant, Cell and Environ.* **37**, 2325–2338 (2014).
